# *Plukenetia volubilis* leaves as source of anti-*Helicobacter pylori* agents

**DOI:** 10.3389/fphar.2024.1461447

**Published:** 2024-10-23

**Authors:** Aditya Tan, Katia Castanho Scortecci, Nathalia Maira Cabral De Medeiros, Wirginia Kukula-Koch, Thomas J. Butler, Sinéad Marian Smith, Fabio Boylan

**Affiliations:** ^1^ School of Pharmacy and Pharmaceutical Sciences, Trinity College Dublin, Trinity Biomedical Sciences Institute, Dublin, Ireland; ^2^ Laboratório de Transformação de Plantas e Análise em Microscopia (LTPAM), Departamento de Biologia Celular e Genética, Federal University of Rio Grande do Norte (UFRN), Natal, Brazil; ^3^ Programa de Pós-Graduação em Bioquímica e Biologia Molecular, Centro de Biociências, UFRN, Natal, Brazil; ^4^ Laboratório de Biotecnologia Vegetal (LBV), Departamento de Biologia, Centro de Ciências Biológicas e da Saúde, Universidade Estadual da Paraiba (UEPB) Campina Grande, Paraiba, Brazil; ^5^ Department of Pharmacognosy With Medicinal Plants Garden, Medical University of Lublin, Lublin, Poland; ^6^ Department of Clinical Medicine, School of Medicine, Trinity College Dublin, Trinity Centre, Tallaght University Hospital, Dublin, Ireland; ^7^ Trinity Natural Products Research Centre, NatPro Centre, Trinity College Dublin, Dublin, Ireland

**Keywords:** sacha inchi, euphorbiaceae, HSCCC, ethyl acetate partition, astragalin, antimicrobial, *Helicobacter pylori*

## Abstract

**Introduction:**

*Helicobacter pylori* infection is a major issue worldwide, with widespread prevalence, combined with its link to gastritis, peptic ulcers, gastric cancer, and mucosa-associated lymphoid tissue (MALT) lymphoma. Meanwhile, effectiveness of current treatment protocols is limited by increasing antibiotic resistance and patient compliance issues due to long regimens and side effects. *Plukenetia volubilis*, or sacha inchi, is a valuable source of bioactive molecules. However, studies on its antimicrobial activity, especially against *H. pylori*, are lacking.

**Methods:**

In this study, the anti-*H. pylori* activity of *P. volubilis* leaves water extract was explored using *in vitro* and *in silico* approaches. High-Performance Liquid Chromatography coupled to Electrospray Ionisation and Quadrupole Time-of-Flight Mass Spectrometry (HPLC-ESI- QTOF-MS-MS) analysis of the water extract from the leaves was used to characterise the chemical composition of the plant and allowed identification of some flavonoids, such as astragalin, and some phenolic compounds. Then, high-speed counter current chromatography (HSCCC) was used to fractionate the ethyl acetate partition obtained from the water extract from the leaves.

**Results and Discussion:**

The presence of flavonoids derived from kaempferol was confirmed and astragalin was isolated for the first time in *P. volubilis*. The *P. volubilis* water infusion, ethyl acetate extract and the isolated astragalin exhibited anti-bacterial activity against *H. pylori* J99 and two clinical isolates (e.g., minimum inhibitory concentrations of 0.53, 0.51 and 0.49 μg/mL, respectively, for clarithromycin-resistant clinical isolate SSR366). Then, using molecular docking for potential protein targets for *H. pylori*, it was verified that astragalin could interact with these proteins by *in silico* analysis.

**Conclusion:**

These findings highlight that *P. volubilis* and astragalin produce a bacteriostatic activity against *H. pylori* and may have potential to be used in treatment against *H. pylori*, after further research.

## 1 Introduction

*Plukenetia volubilis*, known commonly as Sacha Inchi or Inca peanut, is a plant belonging to the Euphorbiaceae, subfamily Acalyphoideae, tribe Plukenetieae (Benth.) Hutch., subtribe Plukenetiinae Benth (Del-Castillo et al., 2019; Goyal et al., 2022). It is native to South America, but records have located the spread of these plants in other regions throughout Asia such as Indonesia and Myanmar (Jang et al., 2020; Kodahl and Sørensen, 2021; Tianara et al., 2024). Currently, this plant is a valuable source of essential oils, and for that reason it is cultivated industrially in the regions of Loreto, San Martín, Lamas, Moyobamba, and El Dorado of tropical America, as well as overseas throughout Asia.

The oil is known to be rich in α-linolenic acid (ALA, omega 3) and beneficial omega- 3:omega-6 balance, protein content, and other bioactive compounds. However, other parts of the plant such as fruit, leaves, and seeds also have bioactive compounds that have beneficial effects upon consumption (Kim and Joo, 2021; Kittibunchakul et al., 2022a; Kittibunchakul et al., 2022b; Lin et al., 2022a). These compounds include saturated and unsaturated fatty acids, tocopherols, phytosterols, polyphenols, sugar molecules, proteins, acids, vitamins and more - many of which are present only in trace amounts and visualised only through comprehensive metabolomic studies (Keawkim and Na Jom, 2022).

Due to its high variety of bioactive compounds, *P. volubilis* is a plant with significant pharmacological properties. Past studies have shown that oil and extract have significant antioxidant, antimicrobial, anti-dyslipidemic, anti-cancer, anti-inflammatory, anti-obesity, neuroprotective, and probiotic-stimulating activities (Kim and Joo, 2021; Nascimento et al., 2013; Rojanaverawong et al., 2023; Srichamnong et al., 2018; Wang et al., 2018). Some of these activities can be attributed to the presence of flavonoids. For instance, a number of flavonoids have been shown to correlate with the DPPH activity, an important antioxidant assay that can be used to predict antioxidant properties based on flavonoid content (Wuttisin et al., 2021a). It has been observed in diabetic rats that *P. volubilis* extracts can have an anti-hyperglycemic action via improvement of glucose-metabolising enzymes, possibly due to its flavonoid composition (Abd Rahman et al., 2023). Considering the flavonoid composition described in the literature for *P. volubilis* (Ramos-Escudero et al., 2021; Kittibunchakul et al., 2022a; Kittibunchakul et al., 2023; Poomanee et al., 2024), the extracts obtained from leaves and fruit capsules may be an excellent source for antimicrobial flavonoids, but more research should be done using different bacteria, for example, *Helicobacter pylori (H. pylori)*.

*H. pylori*, a member of Epsilonproteobacteria, is a gram-negative microaerophilic flagellated bacterium that infects the stomach mucosa (FitzGerald and Smith, 2021). *H. pylori* has been observed in both adults and in children, and usually patients are asymptomatic (Mišak et al., 2019; Roszczenko-Jasińska et al., 2020). However, *H. pylori* infection is associated with stomach diseases, such as gastritis (also known as chronic inflammation of the gastric mucosa), peptic ulcer disease and in some cases gastric cancer and mucosa-associated lymphoid tissue (MALT) lymphoma (Roszczenko-Jasińska et al., 2020; Sun et al., 2023). Countries where *H. pylori* infection was reduced or eradicated also observed a reduction in gastric cancer incidence, showing the correlation of this agent to gastric cancer (Sun et al., 2023). As such, the International Agency for Research on Cancer has classified *H. pylori* as a class I carcinogen (IARC Working Group on the Evaluation of Carcinogenic Risks to Humans, 1994). In addition, the bacteria can also induce iron deficiency anaemia and vitamin B12 deficiency (Howden et al., 2022).

*H. pylori* infect almost half of the global population overall, with the prevalence ranging according to population and country (Li et al., 2023). *H. pylori* has been observed at high frequency in developing countries, due to the economic and health situation of the population. Its prevalence is around 80% or more in adults in Africa, followed by Latin America (63.4%) and Asia (54.7%) (Smith et al., 2018; Sun et al., 2023). *H. pylori* can be spread directly from one person to another or indirectly by the environment (Roszczenko-Jasińska et al., 2020).

The pathogenicity of *H. pylori* infection has been mainly associated with bacterial genotype, host genetic polymorphisms, and inflammatory response (Roszczenko-Jasińska et al., 2020; Kim and Wang, 2021; Sun et al., 2023). The two main bacterial determinants of *H. pylori* pathogenicity are the cytotoxin-associated gene A (CagA) encoded by the cag pathogenicity island (*cag* PAI) and the vacuolating cytotoxin gene A (VacA) (FitzGerald and Smith, 2021). In strains where the *cag* PAI is present, a type IV secretion system is encoded that enables translocation of the CagA protein directly into host cells leading to dysregulation of host intracellular cell signalling pathways. The *vacA* gene is present in all *H. pylori* strains, with different polymorphisms within the *vacA* gene influencing the cytotoxicity of the encoded VacA protein (FitzGerald and Smith, 2021). Numerous studies have shown that strains producing CagA and the more virulent VacA forms are associated with increased risk of severe gastritis, MALT lymphoma and adenocarcinoma of the stomach (Atrisco-Morales et al., 2018, Malfertheiner et al., 2023; Matos et al., 2013; Salimzadeh et al., 2015; Umit et al., 2009).

In terms of bacterial colonisation, the urease gene is an important enzyme that promotes *H. pylori* survival in the acid stomach environment and, in combination with a chemotaxis system, aids the bacteria in reaching the protective mucus layer of the gastric mucosa for efficient infection (Lertsethtakarn et al., 2011; Sycuro et al., 2012; Salillas and Sancho, 2020). Urease has two subunits, UreA and UreB, and for its activity it requires the presence of Nickel ions (Ni) via the nickel-responsive regulator (NikR). Then, it is transported to the cytoplasm by the nickel-cobalt transporter (NixA), where the urease is located (Salillas and Sancho, 2020). For colonisation, the outer-membrane proteins (OMPs) also have an important role in bacterial adhesion to gastric cells; the *oipA* gene encodes the OipA outer membrane protein, and the *babA* gene encodes the BabA protein (Roszczenko-Jasińska et al., 2020; Salillas and Sancho, 2020).

*H. pylori* treatment comprises a proton pump inhibitor (PPI) and 2–3 antimicrobials. In areas of low clarithromycin resistance, the recommended first-line treatment is a clarithromycin, amoxicillin and PPI triple therapy. In areas of high or unknown clarithromycin resistance, a bismuth quadruple therapy is recommended (PPI, bismuth, metronidazole, tetracycline) (Malfertheiner et al., 2022). However, treatment fails in more than 20% of patients nowadays, mainly due to *H. pylori* resistance to antibiotics and also due to patient non-compliance as the treatment may be considered long; from 10 to 14 days according to the protocol used. The World Health Organisation (WHO) (2017) elected *H. pylori* as one of 12 priority pathogens due to antibiotic resistance and the disease severity as a class I carcinogen. As a result of this, it is extremely important to develop new antibiotics and new protocols for *H. pylori* treatment (World Health Organization, 2017; Roszczenko-Jasińska et al., 2020; Liou et al., 2020), and for that reason plants and natural products could potentially play an extremely important role. Potential therapeutic targets include key *H. pylori* proteins involved in bacterial virulence, colonisation and adhesion, such as the above-mentioned CagA, urease, NikR and BabA. Other potential targets include the homeostatic stress regulator (hsrA) sequence, an orphan gene present only in Epsilonproteobacteria. It was verified that the HsrA protein self-regulates and also regulates the expression of different proteins associated with transcription, translation, energy metabolism, redox metabolism, and oxidative stress defence (Salillas and Sancho, 2020). Due to different protein regulations, flavodoxin has been proposed as an ideal marker for drug development. Flavodoxin is a small electron transfer protein that has been verified to be important for bacteria metabolic pathways. It is expressed in *H. pylori* and other gastrointestinal pathogens as well as in human gut commensal bacteria (Salillas and Sancho, 2020; Sallilas et al., 2021).

The benefits of using medicinal plants as a pharmacological aid have been increasingly recognised by governmental agencies. For example, in 2018, 109 countries formed a regulatory framework for traditional medicines, either integrated into the national drug laws or as a standalone framework (World Health Organisation, 2019). In line with governmental efforts, research has also been done to verify the efficacy and safety of medicinal plant use and, in some cases, to improve the bioavailability of the bioactive compounds. Most of the pharmacological activities are related to antioxidant (Bahar et al., 2016), anti-thrombosis, anti-hypertensive (Gao et al., 2016), anti-inflammatory, and antimicrobial (Siritapetawee et al., 2018).

With this in mind, this study aimed to (i) perform a chemical study of *P. volubilis* water extract by HPLC-ESI-QTOF-MS/MS, (ii) separate its ethyl acetate extract by High-Speed Counter Current Chromatography (HSCCC), (iii) perform pharmacological evaluation of the water extract, its ethyl acetate fraction and isolated compound astragalin against *H. pylori,* and (iv) perform molecular docking to investigate binding of the *P. volubilis* isolated flavonoid astragalin to candidate *H. pylori* target proteins.

## 2 Materials and methods

### 2.1 Chemicals

Solvents used for partitioning the water extract of *P. volubilis* (ethyl-acetate) and chromatographic methods were of HPLC-grade quality from SigmaAldrich (Arklow, Ireland). Ultra-pure water was obtained from Millipore™. Sephadex^®^ LH-20 and Methanol-D4 (≥99.8% purity) for Nuclear Magnetic Resonance (NMR) analysis was purchased from SigmaAldrich (Arklow, Ireland).

### 2.2 *P. volubilis* water *extract preparation*

For the extraction process, leaves of *P. volubilis* were collected in June 2023, in the city of Parnamirim-RN (−05^∘^ 47“42'” + 35^∘^ 12“34”), Brazil. The collection was carried out with the licence from the Biodiversity Authorization and Information System (SISBIO) with registration number 70956, SIGEN A39FD4C (Brazil). The species was identified by the botanist from UFRN, Dr. Leonardo de Melo Versieux, and a voucher specimen was deposited in the herbarium of the Federal University of Rio Grande do Norte-UFRN, with the registration number UFRN 10854.

A water extract used in the study was an infusion prepared by steeping 50 g of leaves in 1,000 mL of boiling distilled water (100°C). The flask was covered with aluminum foil to protect it from light and left for 30 min. Afterward, the infusion was filtered using Whatman No. 1 filter paper and concentrated into a powder using a freeze dryer for transport to Trinity College Dublin (TCD).

At TCD, this infusion was resuspended in water, and a partitioning process was carried out using ethyl acetate (EA) in 10 portions of 100 mL each aiming the isolation of phenolic compounds. The EA fraction was then dried using a rotary evaporator before undergoing chemical analysis.

### 2.3 Chemical analysis of P. volubilis EA extract using high-speed counter current chromatography (HSCCC) fractionation

The HSCCC machine used was an *IntroPrepTM (Quattro) (AECSQuikPrep, Cornwall, UK),* which is composed by a column of Polytetrafluoroethylene PTFE tubing with a diameter of 2.0 mm diameter and column of 136 mL, which is turned around a bobbin. Separations occurred at room temperature with 865 rpm (rotation speed using a centrifugal force). The manual sample loop 187.1 mg of sample dissolved in 6 mL of solvent system.

#### 2.3.1 Choice of the solvent system for HSCCC fractionations

The choice of solvent system is a crucial step before the separation using HSCCC. Three solvent systems containing: hexane, ethyl acetate, methanol, and water H:E:M:Wat (1:4:1:4 and 1.5:3.5:1:4) (v/v/v/v); hexane, ethyl acetate, butanol, methanol, and water H:E:B:M:Wat (1:4:0.5:0.5:4) (v/v/v/v/v); ethyl acetate, butanol, and water E:B:Wat (4:1:5 and 3.5:1.5:5) (v/v/v) were tested for the EA extract of *P. volubilis*.

This step was done according to Alsharif et al. (2023), where approximately 2 mg of EA extract was dissolved in separate test tubes containing one of the tested solvent systems described above. Then, these test tubes were shaken and rested until the solvent system separated. Equal aliquots of each phase were spotted on silica gel TLC plates (60,778-25 EA – Sigma Aldrich, Arklow, Ireland). For the TLC run, a mobile phase consisting of ethyl acetate: formic acid: acetic acid: water (10:0.5:0.5:0.5) was used. The plate was sprayed with the natural products reagent (NP/PEG) and visualized under UV light (365 nm). The solvent system used for this study was selected according to the distribution of the compounds in both the upper and lower phases of the biphasic solvent system. For the HSCCC separation it was used for In the case of EA extract, H:E:M:Wat (1.5:3.5:1:4) (v/v/v/v) was selected as the best solvent system to perform the separation.

#### 2.3.2 Preparation of the solvent system for HSCCC

For solvent system preparation, the solvents were added in a separation funnel at room temperature, mixed and allowed to equilibrate. After that, the two-phase formed were separated and collected in different bottles and degassed by sonication for 5 min. The sample solution was prepared by dissolving the sample (0.2 g of EA extract) in the solvent system used for HSCCC separation (aqueous lower phase and organic upper phase (1:1 v/v).

#### 2.3.3 HSCCC separation

The separation was performed in reverse mode, the upper organic layer was used as the stationary phase and the aqueous layer was used as the mobile phase. The HSCCC column was first filled with the stationary phase, using a flow rate of at 2.0 mL/min for the mobile phase into the column, and 860 rpm rotation for HSCCC at room temperature of 25°C. The sample solution (0.2 g of EA extract was solubilised using a 6 mL biphasic system). Sample was injected after the system reached the hydrodynamic equilibrium (Alsharif et al., 2023). Fractions with 4 mL were collected every 2 min for both the elution and the extrusion mode in 10 mL test tubes resulting in 110 fractions (60 tubes for elution and 50 tubes for extrusion).

#### 2.3.4 Purification of isolated compounds

The collected fractions were analysed using TLC plates (Sigma-Aldrich, Arklow, Ireland). Samples with similar elution patterns and retention factors (Rf) were grouped together, and another TLC was performed to analyze the pooled fractions. A semi-purified fraction was submitted to a column Sephadex LH-20 obtained from (Sigma-Aldrich, Arklow, Ireland), for size exclusion. The column was eluted with methanol at an average flow rate of 0.5–1 mL/min. This allows for the full purification of astragalin.

#### 2.3.5 NMR identification

To determine the chemical structure of the isolated compound 1, an NMR analysis was performed, which allowed us to identify the flavonoid astragalin. Astragalin had its structural elucidation using a Bruker Avance 400 instrument, at 400 MHz for proton (^1^H NMR) magnetic resonance and 100 MHz for carbon magnetic resonance (^13^C NMR). Spectral analysis was performed using the Mestrenova software. Chemical shifts are reported as (ppm) values, and the coupling constants are given in Hz. Two-dimensional measurements (^1^H–^1^H COSY, HMBC, HMQC) were obtained on the same instrument.

#### 2.3.6 HPLC analysis

After its separation by HSCCC and structural elucidation using NMR, astragalin was further evaluated using HPLC to assess its purity. Waters HPLC system with Water Alliance 2,695 fitted with Water 2,487 dual λ absorbance detector, a 717 plus autosampler and in line degasser system was used for the analysis. Data was collected using Empower Software. The analytical column used was LiChrospher RP-18 HPLC Column (25 cm × 4.6 mm). The mobile phase used consisted of 0.25% phosphoric acid in water (v/v) (solvent A) and 100% methanol (solvent B). Other parameters included: Gradient: 0–5 min–40% solvent B, 5–10 min–55% B, 10–15 min–65% B, 15–20 min–50% B, 20–25 min–30% B, 25–30 min–40% B, flow rate 1 mL/min, time of analysis: 50 min, injector volume 10 μL and detection wavelength at 254 and 280 nm.

### 2.4 HPLC-ESI-QTOF-MS/MS analysis of the *P. volubilis* water extract

An HPLC-MS analysis was performed with the water extract of *P. volubilis* leaves. For this purpose, an Agilent Technologies platform that was composed of an HPLC chromatograph coupled to a mass spectrometer was used. The platform contained a high-performance liquid chromatograph (1,200 Series, Agilent Technologies, Santa Clara, CA, USA) equipped in a degasser, a binary pump, an autosampler, a column thermostat chamber, a UV detector and a Q-TOF mass spectrometer with electrospray ionization that was operated in negative ion mode. The following settings were introduced for the analysis of *P. volubilis* extract: the fragmentor voltage 110 V, CID energy 10 and 20 V, capillary voltage of 3000 V, skimmer voltage 65 V, nozzle voltage 1000V, *m/z* range of 40–1,200 Da, gas temperature of 250, sheath gas temperature of 300°C, gas flows of 12 L/min, and nebulizer pressure of 35 psi. The gradient of acetonitrile with 0.1% formic acid (solvent B) in 0.1% formic acid in water (solvent A) was used in the program: 0 min: 1% B, 10 min: 20% B, 15 min: 40% of B, 17–18 min: 95% of B, 19–25 min: 1% of B. The run lasted 35 min, the flow rate was set at 0.2 mL/min and the temperature of the thermostat was 20°C. The analysis of data was performed using the Mass Hunter Workstation program (version B.10.00 by Agilent Technologies).

### 2.5 Pharmacological analysis of *P. volubilis* water extract, EA extract and astragalin against *H. pylori*

Two clinical strains of *H. pylori* (SSR359 and SSR366) isolated at Tallaght University Hospital, Dublin were used in this study, as well as the whole-genome sequenced reference strain J99 (American Type Culture Collection 702,824). SSR359 and J99 are clarithromycin sensitive. SSR366 is resistant to clarithromycin with a minimum inhibitory concentration (MIC) of 16 mg/L, which falls well above the clarithromycin resistance breakpoint of 0.25 mg/L defined by the European Committee on Antimicrobial Susceptibility Testing (2024). *H. pylori* cultures were maintained on Columbia blood agar plates containing 5% sheep’s blood (VWR) at 37°C under microaerobic conditions (CampyGen, Oxoid). Bacteria were inoculated into T25 tissue culture flasks with vented lids (Corning), containing Brain Heart Infusion (BHI) broth (Sigma) with 10% foetal bovine serum (FBS; Gibco). The flasks were placed in airtight containers with a CampyGen sachet to generate microaerobic conditions and incubated at 37°C with shaking (180 rpm) for 24 h. The MIC of the *P. volubilis* water extract, the EA extract and astragalin against *H. pylori* was determined using broth microdilution assays in 96-well plates with low evaporation lids (Corning). The water extract, EA extract and astragalin were initially reconstituted in DMSO (Sigma) and subsequently serially diluted in the 96-well plates using BHI broth supplemented with 10% FBS in triplicate. The concentrations tested ranged from 256 - 0.001 μg/mL, which is the concentration range used in routine clarithromycin antimicrobial susceptibility testing for *H. pylori* (Megraud et al., 2013). 100 μL aliquots of bacterial suspension (optical density at 600 nm (OD600)=0.1, corresponding to approximately 1 × 10^8^ colony forming units/mL) were then added to each well of the 96-well plates for a final concentration of 200 µL/well. Untreated bacterial wells were used as a positive control (100% growth) and uninoculated medium was used as a negative growth control (0%). A clarithromycin control was used as a positive experimental control on each plate. The 96-well plates were incubated in airtight containers for 72 h at 37°C under microaerobic conditions with shaking (180 rpm). Absorbances at wavelength 600 nm were read using a Varioskan LUX Microplate Reader (Thermo Fisher Scientific).

Triplicate results were analysed using GraphPad Prism version 10.1.0. Positive and negative control wells were used to calculate the normalised % growth for each concentration of extract tested. The MIC is the lowest concentration that completely inhibits bacterial growth (Lambert and Pearson, 2000). MIC values of test extract required to inhibit the growth of *H. pylori* were determined using the Gompertz fit model (Lambert and Pearson, 2000) on the growth curves generated in GraphPad from the broth microdilution assays.

Minimum bactericidal concentration (MBC) was measured via subsequent transfer of 10 µL of assay culture from each well into a new 96 well plate containing fresh culture medium. Following incubation for 72 h at 37°C under microaerobic conditions with shaking, the lowest concentration at which no growth was observed no growth was defined as the MBC.

### 2.6 Analysis of the effects of *P. volubilis* water extract, EA extract and astragalin on the viability of human gastric epithelial cells

To assess the effect of *P. volubilis* water extract, the EA extract and astragalin on host cell viability, the MTT (3-(4,5-dimethylthiazol-2-yl)-2,5-diphenyltetrazolium bromide) assay was used. AGS human epithelial gastric adenocarcinoma cells (ATCC CRL-1739), with a passage number <20, were maintained in Ham’s F12 nutrient mix (Sigma) supplemented with 10% FBS and cultured in a 5% CO2 humidified 37°C incubator. For MTT assays, 100 μL of AGS cells were seeded into 96-well plates at a concentration of 10,000 cells per well in Ham’s F12 nutrient mix (Sigma) supplemented with 10% FBS. The following day, culture medium was removed and replaced with 100 μL of fresh culture medium containing serial dilutions of the tea, EA extract and astragalin (256 - 0.008 μg/mL) in triplicate. Positive control wells (cell culture medium with 0.1% Triton-X100) and untreated negative control wells (cell culture medium only) were included on each plate. Following incubation, medium was gently removed from each well. MTT was then added to the cells at a concentration of 0.5 mg/mL in cell culture medium and the cells incubated in a 5% CO_2_ humidified 37°C incubator for 2 h. Cell culture medium containing MTT was removed and the insoluble formazan crystals were resuspended via the addition of 100 μL of DMSO to each well. Absorbances at 570 nm were measured on the Varioskan Lux microplate reader. Normalized viability was calculated using the positive control (0%) and negative control (100%) for each concentration of extract tested. Triplicate results were analysed in GraphPad Prism version 10.1.0 and compared using a two-way ANOVA with Sidak’s multiple comparison tests. A p < 0.05 was considered statistically significant.

### 2.7 Molecular docking

A molecular docking for *H. pylori* proteins was performed with the astragalin ligand using Chimera software (version 1.17.3), specifically with AutoDock Vina tool. The protein structures used in this study were obtained from two sources: experimental structures from the Protein Data Bank (PDB, https://www.rcsb.org/) and predicted models from the AlphaFold database (https://alphafold.ebi.ac.uk/). The PDB provided structures for NikR (2WVB), CagA (4DVY), BabA (4ZH0), and Urease (1E9Z), while predicted models of NikR (AF-025896-F1), HsrA (AF-A0A496FN53-F1), CagA (AF-P55980-F1), BabA (AF-Q17SX4-F1), Urease (AF-P69996-F1), and Flavodoxin (AF-O25342-F1) were sourced from AlphaFold. The ligand, astragalin, was obtained from the PubChem database (PubChem astragalin, Supplementary Table S1). These proteins were chosen as potential markers for drug development based on the reviews from Roszczenko-Jasińska et al. (2020) and Salillas and Sancho (2020).

For each protein, the grid box was manually adjusted to cover a significant region of the receptor protein, allowing a broader exploration of potential binding sites rather than focusing strictly on the active site. This approach enabled a comprehensive evaluation of the molecular interactions between astragalin and the proteins. The specific grid box parameters, including the center coordinates and dimensions for each protein-ligand pair, are detailed in Table 1. The table also includes additional settings such as exhaustiveness, number of binding modes, and energy range, ensuring consistency across all simulations.

**TABLE 1:** Molecular docking parameters for each protein and ligand.

Target protein	Ligand	Grid box center (x, y, z)	Grid box size (x, y, z)	Exhaustiveness	Number of modes	Energy range (kcal/mol)
NikR (AlphaFold: AF-O25896-F1)	Astragalin	7.55, 3.42, −5.61	61.66, 23.90, 39.16	8	9	3
NikR (PDB: 2WVB)	Astragalin	31.16, 22.58, −4.03	39.65, 57.59, 36.86	8	9	3
HsrA (AlphaFold: AF-A0A496FN53-F1)	Astragalin	−5.49, −1.33, 4.60	53.24, 34.91, 58.47	8	9	3
CagA (PDB: 4DVY)	Astragalin	2.12, 22.72, −20.69	102.50, 88.42, 80.17	8	9	3
CagA (AlphaFold: AF-P55980-F1)	Astragalin	−8.81, −2.31, 1.02	110.56, 90.61, 140.38	8	9	3
Urease (PDB: 1E9Z)	Astragalin	132.96, 103.15, 71.68	49.69, 91.48, 73.69	8	9	3
Urease (AlphaFold: AF-P69996-F1)	Astragalin	0.30, 5.78, 0.20	81.85, 31.80, 75.83	8	9	3
BabA (PDB: 4ZH0)	Astragalin	11.32, 30.28, 41.85	87.82, 59.50, 97.46	8	9	3
BabA (AlphaFold: AF-Q17SX4-F1)	Astragalin	−23.47, 18.59, 5.04	92.73, 67.46, 70.38	8	9	3
Flavodoxin (AlphaFold: AF-O25342-F1)	Astragalin	1.44, 3.55, −8.23	61.52, 34.96, 57.44	8	9	3

Among the docking solutions generated, the most energetically favorable binding mode for each protein-ligand pair was selected based on the binding energy score (kcal/mol). Lower (more negative) scores indicate stronger and more favorable interactions between the ligand and the protein. These favorable docking configurations were selected for subsequent analysis. Additionally, the root mean square deviation (RMSD) values were assessed to evaluate the stability and consistency of the ligand poses during docking. RMSD values of zero indicated consistent ligand poses, signifying no significant variation during the docking simulations.

To further explore the molecular interactions, hydrogen bond interactions between astragalin and *H. pylori* proteins were identified using Chimera’s “FindHbond” tool. The parameters for hydrogen bond detection were relaxed slightly, allowing a distance adjustment of 0.4 Å and an angular adjustment of 20°, to account for biologically significant interactions that may not fall within stricter criteria. This detailed analysis of hydrogen bonding provided crucial insights into the potential influence of astragalin on the functionality of the target proteins.

## 3 Results

### 3.1 Chemical analysis of *P. volubilis* water extract

**FIGURE 1:**
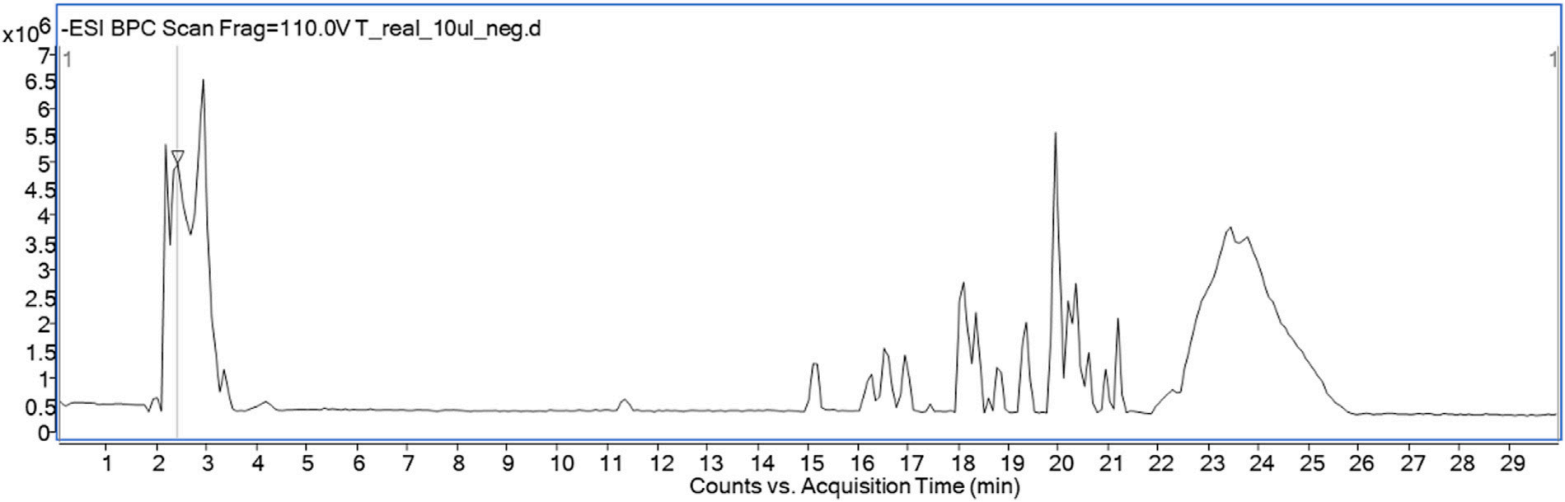
Total ion chromatogram recorded in the negative ionization mode.

Table 2 and Figure 1 show the presence of 16 compounds identified by HPLC-ESI-QTOF-MS/MS in *P. volubilis* water extract. The table shows the identification peaks, retention time, electrospray ionisation mass spectrometry in negative mode and molecular formula for all the compounds detected. The structures were suggested based on the *m/z* of both precursor ion and fragmentation obtained. The spectral data were also compared with that reported in the literature and in the reference metabolome database for the plant (https://www.biosino.org/RefMetaDB). Several flavonoids derived from kaempferol, and quercetin were identified, including astragalin, which was subsequently isolated and spectrally characterised. A previous study with *P. volubilis* suggested the presence of flavonoids derived from kaempferol by a TLC analysis comparing with flavonoid standards (Nascimento et al., 2013).

**TABLE 2:** The list of tentatively identified components of *P. volubilis* water infusion.

No	Ion +/−	Rt [min]	Molecular formula	m/z theoretical	m/z experimental	Error	DBE	MS/MS	Proposed compound
1	-	2.4	C_4_H_6_O_5_	133.0142	133.0131	8.56	2	115.0019 71.0130	Malic acid
2	-	2.3	C_18_H_18_O_9_	377.0878	377.0858	5.3	10	341.1049 197.0183 179.0525	Syringic anhydride
3	-	3.8	C_6_H_8_O_7_	191.0197	191.0204	−3.51	3	129.0252 111.0107 87.0099	Citric acid
4	-	9.3	C_10_H_13_N_5_O_5_	282.0844	282.0857	−4.62	7	210.8985 150.0437 133.0119 88.0381	Crotonoside
5	-	14.75	C_33_H_40_O_21_	771.1989	771.2013	−3.07	14	609.1504 509.0730 446.0863 429.0659 284.0318	Kaempferol trihexoside
6	-	15.07	C_27_H_32_O_6_	451.2126	451.213	−0.86	12	405.2174 289.0722 243.1753	Kushenol D
7	-	15.11	C_27_H_28_O_6_	447.1813	447.1802	2.48	14	401.1870 221.1185 203.1113 179.1045	Crolaevinoid C
8	-	16.7	C_33_H_40_O_21_	771.1989	771.1983	0.82	14	609.1430 446.0824 284.0316 255.0216	Kaempferol trihexoside/kaempferol sophoroside glucoside
9	-	18.0 36	C_27_H_30_O_16_	609.1461	609.1444	2.8	13	446.0798 283.0209 255.0285	Kaempferol dihexoside
10	-	18.82	C_27_H_30_O_16_	609.1461	609.1439	3.62	13	429.0823 284.0308 255.0299 227.0325	Kaempferol dihexoside
11	-	19.7	C_21_H_20_O_12_	463.0882	463.0863	4.09	12	300.0264 271.0206 255.0211	Quercetin glucoside, e.g., isoquercitrin/querceti n galactoside
12	-	20.27	C_21_H_20_O_11_	447.0933	447.0922	2.42	12	284.0323 255.0296 227.0352 211,0407	Kaempferol-3-*O*- glucoside (astragalin)*
13	-	20.604	C_21_H_20_O_11_	447.0933	447.0947	−3.16	12	284.0335 255.0298 227.0328	Kaempferol glucoside
14	-	22.9	C_18_H_28_O_3_	291.1966	291.1960	1.94	5	79.9574	methyl 3-(3,5-di-tert- butyl-4- hydroxyphenyl)propa noate
15	-	23.1	C_17_H_26_O_3_	277.1809	277.1861	−3.17	5	79.9586	Crotonpyrone B
16	-	23.9	C_19_H_30_O_3_	305.2122	305.2123	−0.27	5	209.1537 79.9570	Crotonolide J

Ion, ionization mode negative; Rt, retention time; error, error of measurement in ppm; DBE, double bonds and rings number; MS/MS, *m/z* fragments recorded in the MS/MS, spectra; TR, traced with no MS/MS, spectrum; * - assigned compounds whose structures were later confirmed by NMR).

After the initial analysis of the *P. volubilis* water infusion, an extraction using ethyl acetate (EA) was performed to facilitate the isolation of compounds using the HSCCC apparatus.

Before deciding for a solvent system for the HSCCC separation, a suitable mobile phase for the separation of the compounds on TLC was devised. Five different combinations of solvents were attempted such as: ethyl acetate: formic acid: acetic acid: water (10:1.1:1.1:.5 or 10:2.6:2.6:1 or 10:0.5:0.5:0.5) (v/v/v/v), butanol: acetic acid: water (3:1:4) (v/v/v/v) and methanol: chloroform: water (6.5:3.5:1) (v/v/v/v). The best mobile phase to separate the compounds of the EA extract was ethyl acetate: formic acid: acetic acid: water (10:0.5:0.5:0.5) (v/v/v/v). After the elution, the TLC was sprayed with natural products/PEG reagent (NP/PG), showing the presence of several green-yellow and orange-yellow bands, indicating the presence of flavonoids and some blue bands suggesting the presence of phenolic compounds.

For HSCCC separation, the solvent system for the analysis of *P. volubilis* EA extract had to be investigated. Four combinations of solvents were tried: hexane, ethyl acetate, methanol, and water H:E:M:Wat (1:4:1:4 and 1.5:3.5:1:4) (v/v/v/v); hexane, ethyl acetate, butanol, methanol, and water H:E:B:M:Wat (1:4:0.5:0.5:4) (v/v/v/v); butanol, ethyl acetate, and water E:B:Wat (4:1:5 and 3.5:1.5:5) (v/v/v/v). The upper and lower phases were separated and analysed separately by TLC. The solvent system chosen for the HSCCC separation of theEA extract was H:E:M:Wat (1.5:3.5:1:4) (v/v/v/v).

**FIGURE 2:**
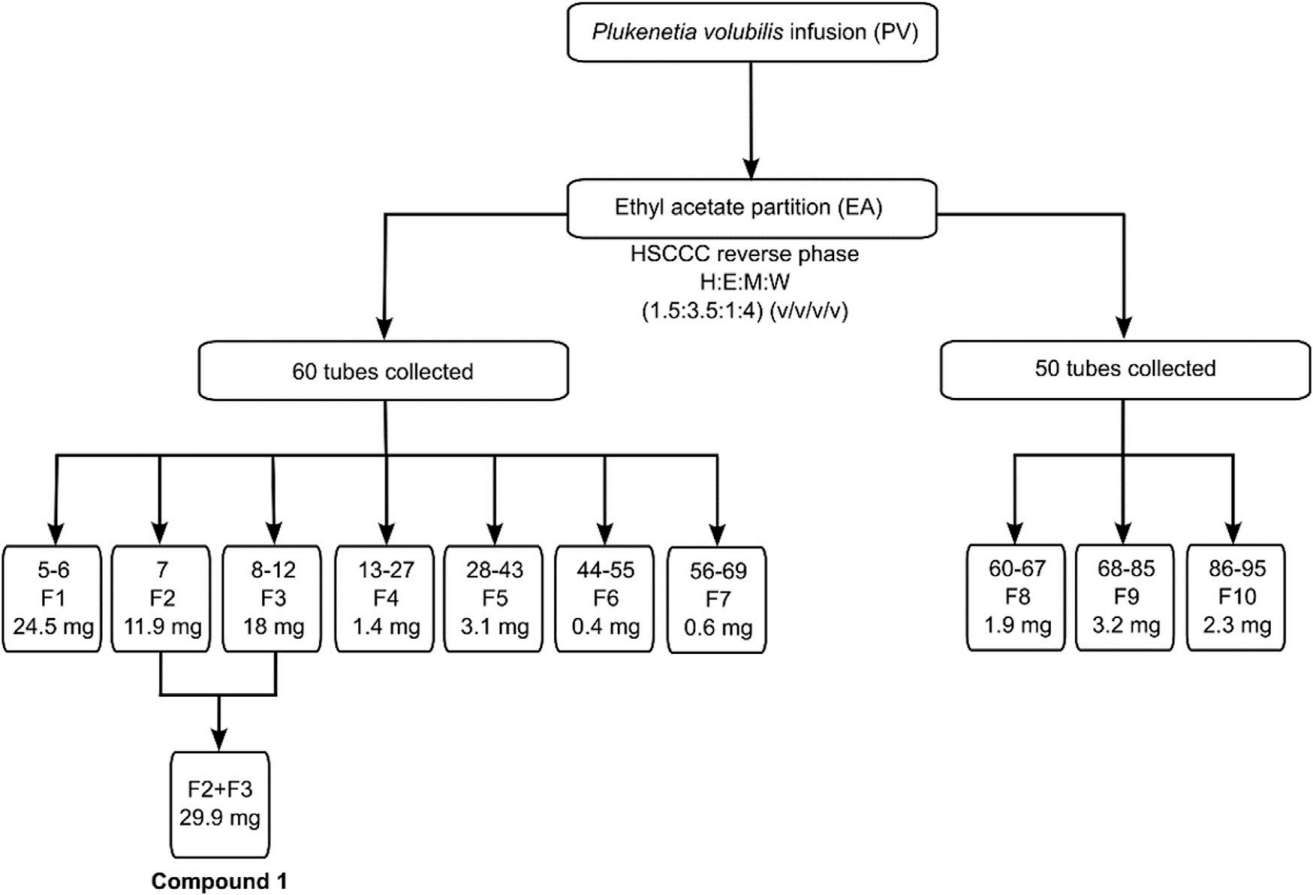
HSCCC fractions obtained with *P. volubilis* EA extract and the isolation of astragalin.

The HSCCC separation for the EA extract was done in reverse mode (head to tail) and the collected samples were pooled into 10 fractions based on their chromatographic similarities after TLC analysis (Figure 2). Fractions 2 and 3 were combined and further purified using Sephadex LH-20. TLC analysis showed that fractions 2 and 3 corresponded to a single compound (F2+F3 – Compound 1), that was further submitted for NMR analysis and characterised as astragalin.

**FIGURE 3:**
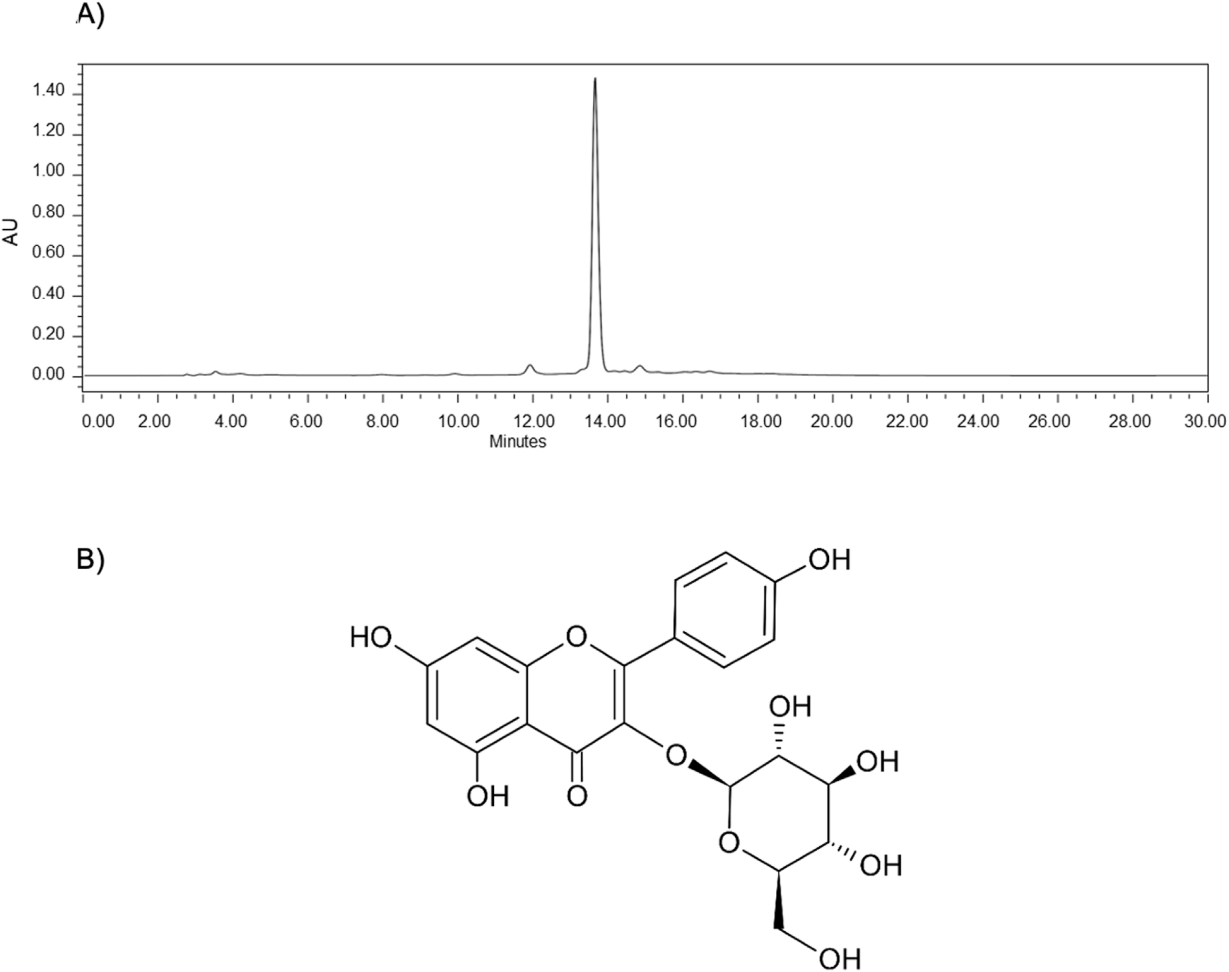
Astragalin isolated from *P. volubilis* EA by HSCCC. **(A)** The HPLC chromatogram to show the purity; **(B)** the structure determined by ^1^D and ^2^D NMR analysis.

The astragalin purity was checked by HPLC-UV analysis (Figure 3A). Astragalin had its structure determined by 1D- and 2D-NMR (Figure 3B), in which they coincided with previous spectroscopic records of NMR collected from *Cedrus brevifolia* A (Douros et al., 2018). The complete NMR spectra are presented in the Supplementary Material (Supplementary Figure S1).

Compound 1 - astragalin (Figure 3B) – yellow: Kaempferol-3-*O*-glucoside; kaempferol-3-glucoside; 480–10–4 (117 mg); ESI−MS for: C_21_H_20_O_11_ molecular ion, *m/z* 447.0933; NMR description - The chemical signals for astragalin are described in ppm as follows: 1H-NMR spectrum (methanol-d4, 400 MHz) are as follows: δ8.04 (d, J = 8.60, 2H), 6.87 (d, J = 8.60, 2H), 6.37 (s, 1H), 6.18 (s, 1H), 5.23 (d, J = 7.20, 1H), 3.69 (dd, J = 12.01, 1.98 Hz, 1H), 3.53 (dd, J = 11.83, 5.32 Hz, 1H), 3.43 (m, 2H), 3.30 (m, 1H), 3.22 (m, 1H). 13C-NMR spectrum (methanol-d4, 100 MHz) are as follows: δ178.10, 164.55, 161.64, 160.17, 157.68, 157.07, 134.08, 130.90, 121.38, 114.68, 104.34, 102.73, 98.50, 93.38, 77.00, 76.64, 74.34, 69.95, 61.23 (^1^D and ^2^D NMR are provided in Supplementary Material–Supplementary Figure S1).

### 3.2 Pharmacological analysis of *P. volubilis* water infusion, EA extract and astragalin against *H. pylori* and AGS cells

**FIGURE 4:**
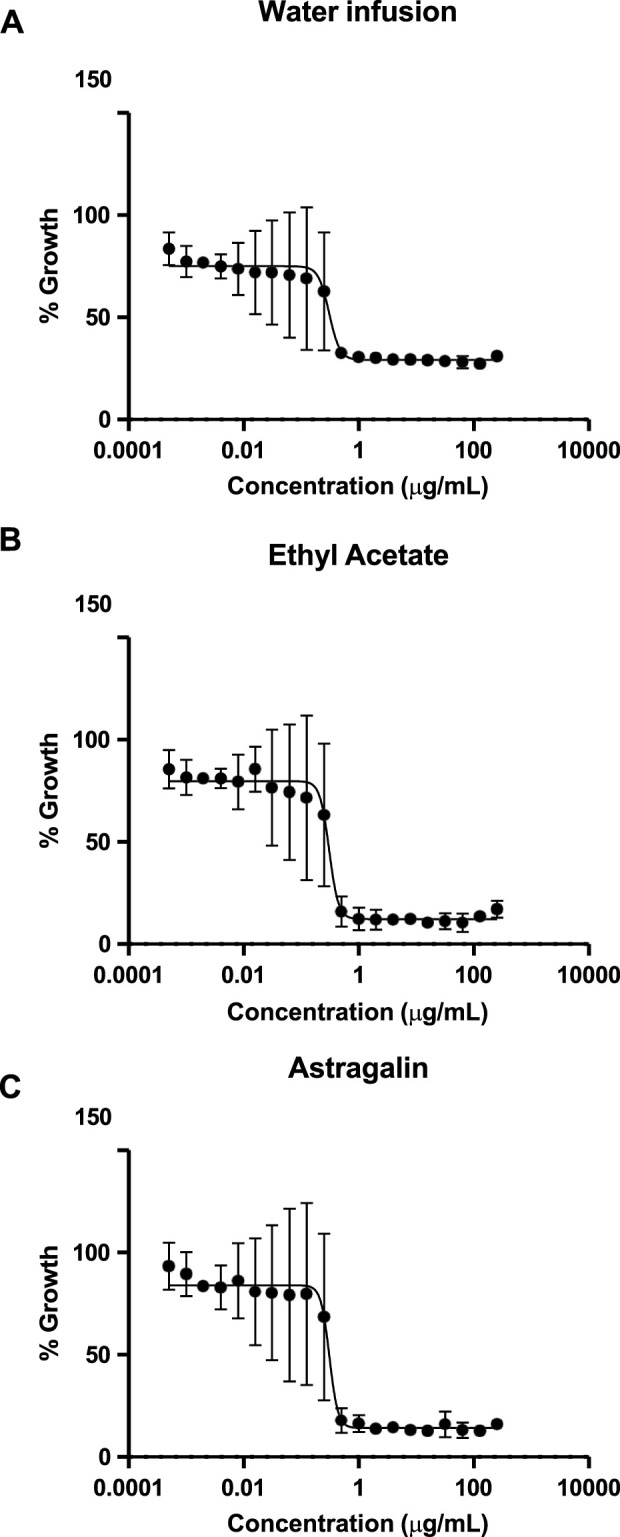
Growth of ***H.*** *pylori* is inhibited by *P. volubilis* water infusion, EA extract and astragalin. Broth microdilution assays were used to expose a clarithromycin-resistant clinical isolate of *H. pylori* (SSR366) to increasing concentrations of *P. volubilis* water infusion **(A)**, ethyl acetate extract **(B)** and astragalin **(C)**. Data points represent mean and standard deviation (error bars) of triplicate values. The curve was generated using non-linear regression. The graphs were then analysed using the Gompertz fit model to determine the MIC values (Table 3).

**FIGURE 5:**
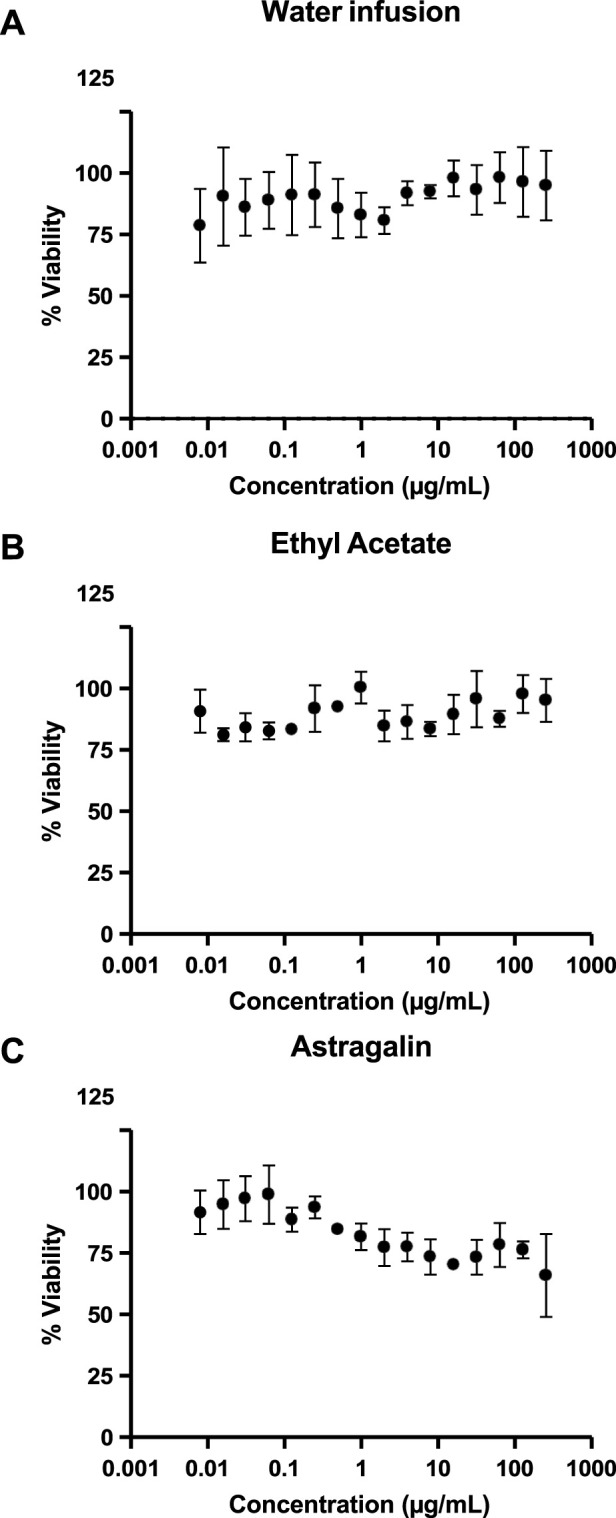
*P. volubilis* water infusion, EA extract and astragalin do not affect the viability of AGS human epithelial cells. AGS cells were treated with increasing concentrations of *P. volubilis* water infusion **(A)**, ethyl acetate extract **(B)** and astragalin **(C)** and analysed following MTT assay. Results are presented as mean and standard deviation (error bars) of triplicate values and were compared using two-way ANOVA with the Sidak’s multiple comparison test. No statistically significant differences were observed.

The antimicrobial activity of *P. volubilis* water infusion, EA extract and astragalin was investigated by exposure to *H. pylori* strain J99 and clinical isolate SSRS359 (both clarithromycin sensitive) and to clarithromycin-resistant clinical isolate of *H. pylori* (SSR366). Similar to the findings of others (Ansorg et al., 1996), DMSO alone had no effect on the viability of the *H. pylori* strains at the concentrations (not shown). Representative growth curves obtained using SSR366 exposed to the water infusion, EA extract and astragalin are shown in Figure 4. All three test substances inhibited the growth of *H. pylori* (Figure 4; Table 2). For the clarithromycin-resistant clinical isolate SSR366, the greatest level of inhibition was observed using astragalin (Figure 4C; Table 3 (MIC 0.49 μg/mL)). High MBCs were obtained using all 3 test substances (Table 3). Additionally, the high ratio of MBC:MIC for each test extract implies that the anti-bacterial properties of the test extracts are bacteriostatic rather than bactericidal (Peterson and Shanholtzer, 1992). To investigate whether these growth inhibitory effects were specific to *H. pylori*, MTT assays were performed using human AGS gastric epithelial cells. None of the test extracts or compounds significantly affected AGS cell viability (Figure 5), demonstrating the selective inhibitory action of the *P. volubilis* extracts against *H. pylori*. DMSO alone did not affect AGS cell viability (not shown).

**TABLE 3:** – Minimum inhibitory- and bactericidal-concentrations of *P. volubilis* water infusion, EA extract and astragalin against *H. pylori*.

	MIC (µg/mL)			MBC (µg/mL)		
*H. pylori* strain	J99	SSR359	SSR366	J99	SSR359	SSR366
*P. volubilis* water infusion	1.18	3.2	0.53	128	256	256
EA extract	0.63	1.5	0.51	64	64	64
Astragalin	1.25	0.5	0.49	256	128	128

MIC, minimum inhibitory concentration; MBC, minimum bactericidal concentration; EA, ethyl acetate.

### 3.3 Molecular docking

Molecular docking using three-dimensional models were used to evaluate possible interactions between key *H. pylori* target proteins and the flavonoid astragalin (ligand) present in *P. volubilis* tea (water infusion) and isolated from its EA extract.

Table 4 presents the results obtained for the molecular docking of the different *H. pylori* proteins (potential targets for treatment according to Roszczenko-Jasińska et al. (2020), Salillas and Sancho (2020) with astragalin ligand. For each entry in the Table 4, a specific protein is identified by name, along with the structural model used in the docking. The structural models mentioned include both structures obtained experimentally and catalogued in the Protein Data Bank (PDB) and structures predicted using the AlphaFold tool. The “Score (kcal/mol)” mentioned in Table 4 represents the binding energy calculated during the docking process, where more negative values indicate more energetically favourable interactions between the protein and the ligand. These results showed that the interaction with astragalin were energetically favourable.

**TABLE 4:** Interaction of astragalin with the different tested proteins.

Protein	E-structure	Score (kcal/mol)	RMSD L.b. (Å)	RMSD U.b. (Å)
NikR	AF-025896-F1	−6.9	0	0
NikR	2WVB	−8.7	0	0
HsrA	AF-A0A496FN53-F1	−7.0	0	0
CagA	AF-P55980-F1	−7.6	0	0
CagA	4DVY	−8.7	0	0
Urease	AF-P69996-F1	−8.5	0	0
Urease	1E9Z	−7.7	0	0
BabA	AF-Q17SX4-F1	−6.2	0	0
BabA	4ZH0	−7.6	0	0
Flavodoxin	AF-O25342-F1	−7.7	0	0

Furthermore, RMSD L.b. (Å) and RMSD U.b. (Å) values, which represent the Root Mean Square Deviation at the lower and upper limit, respectively, are used to assess the structural variation between the different poses of the ligand during docking. In Table 4, both RMSD values are zero, indicating that there was no significant variation between the astragalin ligand poses calculated for each structure. This consistency suggests a reliability in the binding poses obtained for each protein-ligand configuration analysed.

**FIGURE 6:**
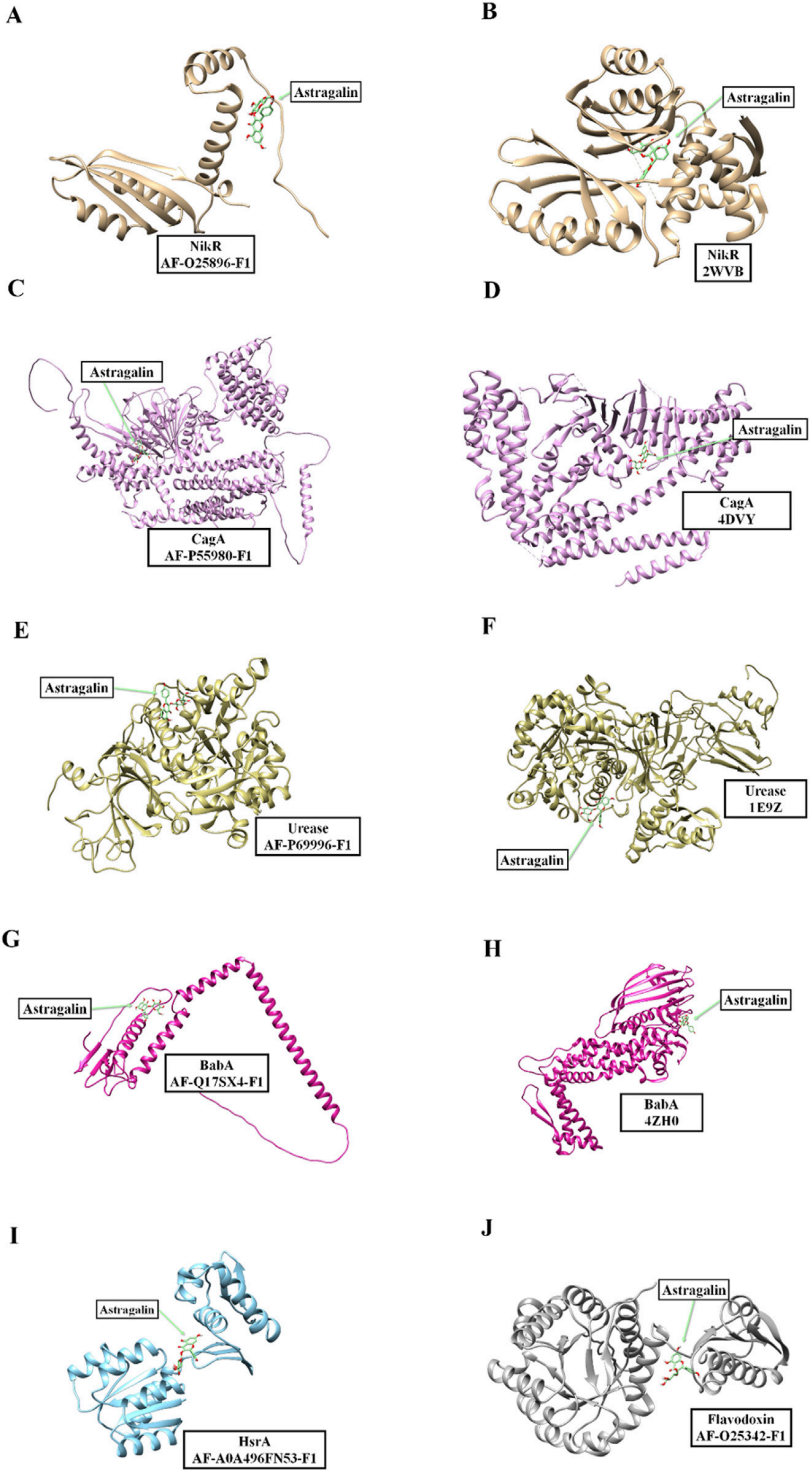
Visualizations of the molecular interactions between *H. pylori* proteins and astragalin. Detailed representations of the interactions between astragalin ligand and various *Helicobacter pylori* proteins. The structures are derived both from crystal data from the Protein Data Bank and from models predicted by the AlphaFold platform. The images specifically illustrate astragalin’s interactions with: NikR Panels **(A, B)**, CagA Panels **(C, D)**, Urease Panels **(E, F)**, BabA Panels **(G, H)**, HsrA Panel **(I)**, Flavodoxin Panel **(J)**. The proteins are coloured according to their structural representation, highlighting the regions of interaction with astragalin ligand, marked in green.

Figure 6 shows a series of visualizations of the molecular interactions between the various *H. pylori* proteins and the astragalin ligand resulting from molecular docking analysis. The structures analysed include both crystal data obtained from the Protein Data Bank and structural models predicted by the AlphaFold platform. These visualizations elucidate the binding sites and offer valuable insights into how astragalin can modulate the function of these proteins, potentially influencing their biological activities within the bacterial cell. This set of images not only highlights the structural variability of the proteins involved, but also underlines the importance of studying such interactions for the development of targeted therapeutic strategies against the pathogenicity of *H. pylori*.

Table 5 shows the results of the molecular docking analysis, which investigated the hydrogen bond interactions between the various *H. pylori* proteins and astragalin ligand. This analysis provides detailed information on the residues involved, the donor and receptor atoms, and the hydrogen atoms that participate directly in the bridges. The distances between the donor and the acceptor (D.A Distance) and between the hydrogen and the acceptor (D-H.A Distance) are documented, with values varying approximately between 2.0 Å and 3.3 Å. These distances are indicative of the strength and potential stability of these interactions. The formation of hydrogen bonds is essential, as it can significantly alter the conformation of the proteins involved, directly affecting their biological function and ability to interact with other biomolecules or substrates. Understanding these interactions is fundamental to elucidating the mechanisms by which astragalin can affect the activities of *H. pylori.*

**TABLE 5:** ***H. pylori*** protein hydrogen bond analysis with astragalin ligand**.** This table presents the detailed results of the hydrogen bond interactions between various *H. pylori* proteins and the astragalin ligand, obtained through molecular docking simulations. The information is organized as follows: Residue Info (Model): Information about the residue and the protein model, including the protein identifier and its origin (PDB or AlphaFold). Atom Donor (Model): Hydrogen donor atom within the protein-ligand complex, including the identification of the protein it comes from. Atom Acceptor (Model): Hydrogen acceptor atom in the astragalin ligand, showing direct interaction with the protein. Hydrogen Atom: Hydrogen atom involved in the bridge, facilitating the bond between donor and acceptor. D.A Distance (Å): Distance between the donor and acceptor atom (D.A), measured in angstroms (Å), indicative of the physical proximity required for an effective hydrogen bridge. D-H.A Distance (Å): Distance between the hydrogen of the donor atom and the acceptor atom (D-H.A), also measured in angstroms, providing details on the spatial orientation of the interaction.

Residue Info (Model)	Atom Donor (Model)	Atom Acceptor (Model)	Hydrogen Atom	D..A Distance (Å)	D-H..A Distance (Å)
ARG 12.A (NikR/AF-O25896- F1)	NE (NikR/AF- O25896-F1)	O11 (astragalin)	HE	3.238	2.310
ARG 12.A (NikR/AF-O25896- F1)	NH2 (NikR/AF- O25896-F1)	O11 (astragalin)	HH21	3.193	2.278
PHE 13.A (NikR/AF-O25896- F1)	O (NikR/AF- O25896-F1)	O6 (astragalin)	H11	3.142	2.200
LYS 140.A (NikR/2WVB)	NZ (NikR/2WVB)	O6 (astragalin)	HZ3	3.053	2.272
LEU 138.A (NikR/2WVB)	O (NikR/2WVB)	O6 (astragalin)	H11	3.031	2.082
HIS 66.A (HsrA/AF- A0A496FN53-F1)	ND1 (HsrA/AF- A0A496FN53-F1)	O3 (astragalin)	H8	3.331	2.372
SER 68.A (HsrA/AF- A0A496FN53-F1)	OG (HsrA/AF- A0A496FN53-F1)	O6 (astragalin)	H11	2.930	2.222
VAL 1111.A (CagA/AF- P55980-F1)	O (CagA/AF- P55980-F1)	O4 (astragalin)	H9	2.990	2.196
TYR 508.A (CagA/AF- P55980-F1)	O (CagA/AF- P55980-F1)	O9 (astragalin)	H18	2.959	2.114
ASN 411.P (CagA/4DVY)	OD1 (CagA/4DVY)	O4 (astragalin)	H9	3.170	2.261
GLU 313.A (urease/AF- P69996-F1)	OE2 (urease/AF- P69996-F1)	O4 (astragalin)	H9	2.692	1.811
ILE 304.A (urease/AF- P69996-F1)	O (urease/AF- P69996-F1)	O5 (astragalin)	H10	3.383	2.589
ARG 375.B (urease/1E9Z)	NH1 (urease/1E9Z)	O5 (astragalin)	HH12	2.978	2.049
ARG 375.B (urease/1E9Z)	NH2 (urease/1E9Z)	O5 (astragalin)	HH22	3.276	2.462
ASN 189.A (BabA/AF- Q17SX4-F1)	ND2 (BabA/AF- Q17SX4-F1)	O10 (astragalin)	HD22	3.134	2.347
TYR 102.A (BabA/4ZH0)	O (BabA/4ZH0)	O5 (astragalin)	H10	2.967	2.144
THR 134.A (BabA/4ZH0)	O (BabA/4ZH0)	O6 (astragalin)	H11	3.089	2.236
SER 132.A (BabA/4ZH0)	O (BabA/4ZH0)	O9 (astragalin)	H18	2.964	2.084
ARG 246.A (Flavodoxin/AF- O25342-F1)	NE (Flavodoxin/AF- O25342-F1)	O4 (astragalin)	HE	2.787	1.994
ARG 246.A (Flavodoxin/AF- O25342-F1)	NH2 (Flavodoxin/AF- O25342-F1)	O4 (astragalin)	HH21	2.828	2.121
ARG 250.A (Flavodoxin/AF- O25342-F1)	NH2 (Flavodoxin/AF- O25342-F1)	O6 (astragalin)	HH21	3.212	2.357
LEU 285.A (Flavodoxin/AF- O25342-F1)	O (Flavodoxin/AF- O25342-F1)	O11 (astragalin)	H20	2.592	1.844
ALA 50.A (Flavodoxin/AF- O25342-F1)	O (Flavodoxin/AF- O25342-F1)	O3 (astragalin)	H8	3.248	2.604

**FIGURE 7:**
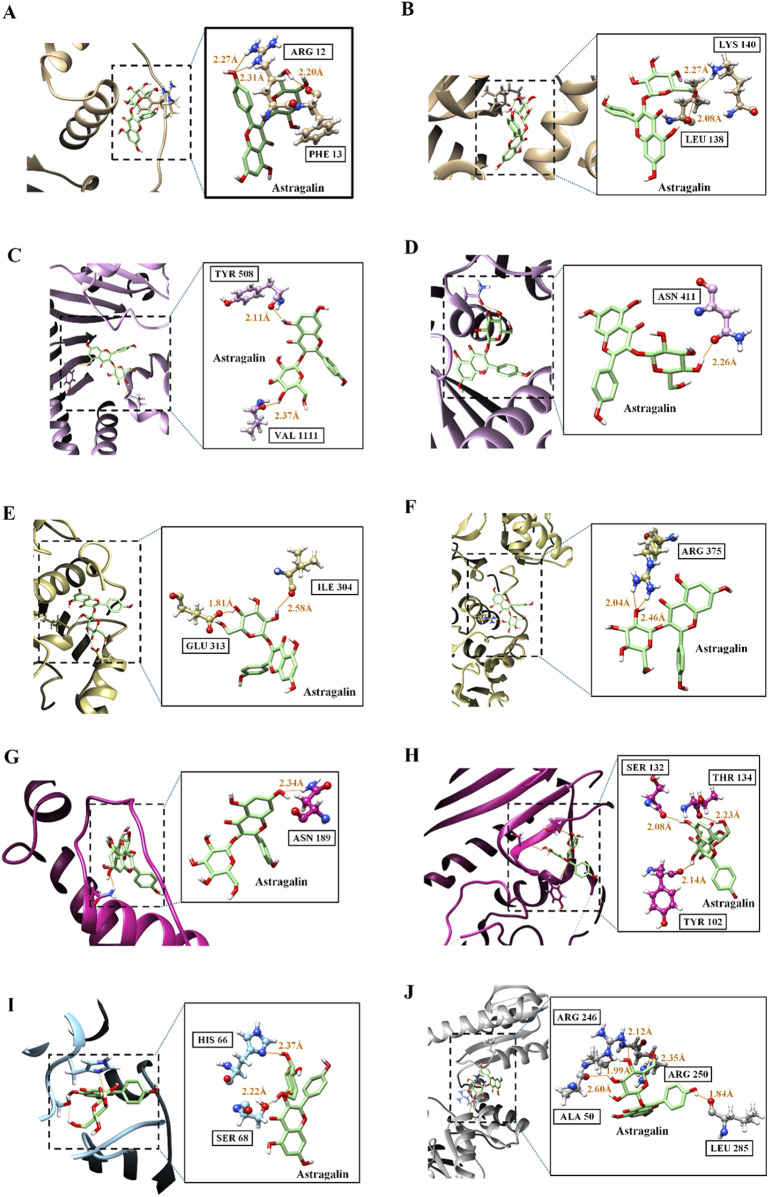
Visualization of hydrogen bond interactions between astragalin and proteins of *H.pylori*. The figure provides a detailed illustration of the hydrogen bond interactions between the ligand astragalin and various proteins of *H. pylori*. This series of panels displays specific visualizations of the protein-ligand complexes, including NikR Panels **(A, B)**, CagA Panels **(C, D)**, Urease Panels **(E, F)**, BabA Panels **(G, H)**, HsrA Panel **(I)**, and Flavodoxin Panel **(J)**. Each panel highlights the interaction sites, where donor and acceptor atoms are represented, and the hydrogen bonds are emphasized in orange, offering a visual understanding of the quantitative measurements provided in the associated table. Green: Carbon atoms, representing the backbone or side chains of the protein and ligand structures. Red: Oxygen atoms, often involved in hydrogen bond interactions as acceptors. Blue: Nitrogen atoms, commonly present in the side chains of nucleotide bases and functional groups that can act as donors in hydrogen bonds. White: Hydrogen atoms, essential in the formation of hydrogen bonds, connecting with oxygen or nitrogen atoms to complete the interaction.

Figure 7 complements the data presented in Table 5, providing a detailed visualisation of the hydrogen bonding interactions between the ligand astragalin and various proteins of *H. pylori*. This set of images specifically illustrates the interactions within the protein-ligand complexes. Each panel offers a detailed graphic representation of the binding sites, highlighting the donor and acceptor atoms, with the hydrogen bonds emphasised in orange. These visualisations not only corroborate the quantitative measurements provided in the Table 5, but also offer a deeper understanding of the spatial nature of these critical interactions.

## 4 Discussion

Medicinal plants have been used for their health benefits since a long time ago. Historical literature from various sources have been unearthed to verify that medicinal plants have been extensively recorded for disease treatment and prevention (Petrovska, 2012). The knowledge of these medicines is seldom documented and passed down orally throughout generations that requires *in situ* observation and conversation with locals of those specific regions to unveil their importance, especially in inaccessible regions (Rajoo et al., 2023; Richard et al., 2023; Mon et al., 2024). Processing of these plants into medicines are usually rudimentary and mainly uses limited equipment, including but not limited to sun-dried, boiled, ground, pounded, chewed, wrapped, pressed, or mixed with foods.

Even with the advent of more modern medicine, these plants are still an important source for alternative treatment for people that prefer to use natural products as a preventive medicine and the others that are suffering drug side effects. In addition, the basic and applied research using these medicinal plants are a source for identification of new bioactive molecules as well as new treatment for old diseases (Yang et al., 2014; Welz et al., 2018). It can be mentioned that roughly a quarter of all FDA- and EMA-approved drugs were obtained from plants, using these bioactive molecules or their derivatives, which shows the importance of them as they take a significant portion of the market (Thomford et al., 2018).

One important class of the bioactive molecules naturally synthesised by plants is polyphenols. This class is formed by phenolics, stilbenes, flavonoids, tannins and ligninoids and are known to have phenolic groups. These compounds are widely found in different plant parts, which are consumed in our diets (e.g., fruits, vegetables, leaves, roots, or seeds), or from their processed extracts (e.g., water extracts like tea and coffee, pressed oils, or other extracts obtained with ethanol or essential oil). As these bioactive compounds are present in our diet, it raises interest in research to correlate the health-benefits related with plant-based diet and the high number of polyphenol content in these products (Pandey and Ibrahim Rizvi, 2009; Spencer et al., 2008).

It has been verified that on consumption, only 5–10 percent of total polyphenols is absorbed by the small intestine to enter systemic circulation, while the remaining can be accumulated in the colon, where they are processed by the gut microbiota to produce other metabolites. In the liver, polyphenols may go through Phase I metabolism consisting of oxidation, reduction, or hydrolysis before proceeding to the Phase II metabolism. In this phase, the hydroxyl groups of polyphenols are subjected to glucuronidation, sulphation, and O-methylation catalysed by the enzymes uridine-5-diphosphate glucuronosyltransferase (UGT), sulfotransferase (SULT) and catechol-*O*-methyltransferase (COMT) (Cassidy and Minihane, 2017).

Furthermore, the fraction that passes to the colon is metabolised by the gut microbiota by their bacterial enzymes, to improve the bioavailability of the metabolites and enhance the benefit of polyphenols to gut health. Interestingly, polyphenols affect the intestinal ecology within this region, in turn supporting the proliferation of probiotic bacteria such as *Bifidobacterium* and *Lactobacillus* and hindering pathogenic bacteria like *Escherichia coli*, *Clostridium perfringens* and *H. pylori* (Bešlo et al., 2023; Zhang et al., 2021). The importance of polyphenols and beneficial bacteria can be observed in this example, where anthocyanins (from grape or wine) can be metabolised into protocatechuic acid in the gut by *Bifidobacterium* and *Lactobacillus* and this allows the proliferation of these bacteria (Man et al., 2020). This example shows that a two-way relationship between polyphenols and the gut microbiota exists (Makarewicz et al., 2021).

Several theories have been proposed to explain the potential action of flavonoids as antimicrobials. One theory is based on the presence of hydroxyl groups which can work as functional sites for radical scavenging and metal ion chelating activity to promote its antioxidant properties (González et al., 2021; Hasnat et al., 2024). Then, this antimicrobial activity of flavonoids can be triggered by the hydroxyl or ketone group, which has the potential to chelate with the nickel (Ni) atom or a residue close by to the nickel atom in the active site of urease enzyme present in bacteria (Biglar et al., 2012; Uddin et al., 2016). Molecular docking done for quercetin showed that the hydroxyl groups that are prevalent were found to be 3-hydroxy, 5-hydroxy, and 3′,4′-dihydroxy groups in flavonoids, where substitution or elimination of any of these groups resulted in a significant reduction in urease inhibitory activity (Xiao et al., 2012). Further research using the molecular docking approach provided the result that quercetin can interact with the catalytic site of urease by binding with the zinc cation (Egas et al., 2018). The importance of the hydroxyl group is also highlighted on the baicalin and scutellarin (two other flavonoids) in their hydrogen binding between the OH group and the flap enclosing the active site of urease (Yu et al., 2015). The urease inhibitory activity is also markedly present in the flavonoids hesperetin and 7-*O*-butylnaringenin (Moon et al., 2013). Furthermore, Saravanakumar et al. (2019) working with extracts from pedicels of persimmon (*D. kaki.* L.) observed that the biomolecules had an anti-*H. pylori* activity. Their analysis showed inhibition of *H. pylori* urease and peptide deformylase, and by molecular docking they were able to verify that these bioactive molecules were able to interact with urea amidohydrolase inhibiting its activity in a synergistic manner. Thus, it has been shown that urease is an essential enzyme for *H. pylori* maintenance in stomach cells (Salillas and Sancho, 2020).

In addition, it has been suggested that hydrophilic flavonoids may act towards the protein surface or cytosolic proteins through the formation of flavonoid-protein complexes to inactivate specific cell functions, including adhesins, cell membrane transporters, transcription

Factors, enzymes, and toxins (González et al., 2021; Górniak et al., 2019). Similarly, lipophilic flavonoids can diffuse into the cell to modulate membrane fluidity and permeability (González et al., 2021; Tarahovsky et al., 2014).

Flavonoids can also have antimicrobial activity from binding to crucial bacterial enzymes. For example, it has been shown that apigenin and quercetin can affect *H. pylori* by binding to the D-alanine:D-alanine ligase (Ddl) as an competitive and non-competitive inhibitor to ATP and D-alanine, respectively (Wu et al., 2008). These two compounds, as well as (S)- sakuranetin, have also shown an inhibitory activity against β-hydroxyacyl-acyl carrier protein dehydratase (HpFabZ) through combined hydrophobic interactions and hydrogen bonding with the tunnel-like structure in HpFabz (Zhang et al., 2008). The other antimicrobial mechanism observed might be via the binding of flavonoids to the effector C-terminal domain of *H. pylori* gene HrsA, consequently forming a HrsA-flavonoid stable complex and then preventing the gene from bind to transcriptional factors. This mechanism has been also observed for flavonoids chrysin, apigenin, hesperetin and kaempferol (González et al., 2019). In addition, hesperetin can also prevented by the activation of virulence factors UreA and UreB, in addition to several genes connected to their flagella motility (Kim et al., 2021).

The observed hydrogen bonds suggest that astragalin may directly alter the function of the NikR protein (Figures 7A, B), interfering with its ability to bind to DNA and interact with other cofactors and regulatory proteins. Such influence is critical, given NikR’s role in regulating metal metabolism and urease expression, key components for the *H. pylori* response to the gastric environment. Furthermore, the interactions also indicate that astragalin may modulate the activity of CagA (Figures 7C, D), a protein associated with the development of gastric carcinoma, a pathology significantly linked to the presence of the CagA gene in *H. pylori* strains.

The analysis of hydrogen bonds between astragalin and urease reveals potential modifications in the functionality of this enzyme (Figures 7E, F), which is crucial for bacterial pathogenesis by catalysing the hydrolysis of urea to produce ammonia (Mobley et al., 2001). Changes in urease activity could compromise the neutralization of gastric acid, essential for bacterial survival in the stomach. Similarly, interaction with BabA (Figures 7G, H), which binds to the fucosylated ABH antigens of the ABO blood group, may reduce the bacterium’s ability to adhere to the gastric mucosa, limiting its colonization and mitigating its pathogenicity (Fujimoto et al., 2007).

Other targets, such gene translocation systems and cell membranes, also are studied for their possible antimicrobial mechanism. It has been observed that kaempferol, for example, can prevents the translocation of *H. pylori* virulence factors VacA and CagA to host cells by preventing the secretion of these factors. Results from the Western blot suggest that the modulation of CagA may be caused by kaempferol-associated decrease of mRNA level of T4SS component, and VacA by inhibition of T5SS secretory genes (Yeon et al., 2019). Similarly, it was shown that hesperetin decreased the expression of critical genes associated to replication and transcription process for *H. pylori* growth (Kim et al., 2021). Other flavonoids may also contribute to bacterial membrane perturbation, as it was verified by the administration of the *Leonotis nepetaefolia* (L.) R. Br hydroethanolic extract. This extract contains 9 types of flavonoids, which simultaneously triggers the increase of membrane permeability, intense elevation of potassium efflux, and nucleotide leakage (Oliveira et al., 2015). Besides membrane destruction, sub-MIC doses of myricetin can also delay the spiral-to-coccoid transition of *H. pylori* (Krzyżek et al., 2021). It has been verified that this inhibition is important to prevent *H. pylori* coccoid-related antibiotic tolerance, caused by the lower level of metabolism, a tendency to self-aggregate and form biofilms, and a higher efflux pump expression of the coccoid form (Krzyżek and Grande, 2020).

Additionally, astragalin interacts with HsrA (Figure 7I), a global regulator that coordinates metabolic and virulence functions. Finally, the interactions with flavodoxin (Figure 7J) might modify its function as an electron acceptor in the pyruvate-oxidoreductase complex, affecting the efficiency of the metabolic decarboxylation reactions of pyruvate, a vital process for bacterial energy production (Cremades et al., 2005).

*P. volubilis* has garnered significant attention in the past few years due to the rich content of omega 3, 6, 9, alpha-tocopherol in its seeds, and it has been verified that its seeds and leaves contains flavonoids and phenolic compounds that have been characterised to have different pharmacological activities as cardiovascular diseases, antioxidant, anti-inflammatory, anti-tumour, antimicrobial, dyslipidaemia and other activities (Wang et al., 2018; Goyal et a., 2022; Kittibunchakul et al., 2023; Rodzi and Lee, 2022; Abd Rahman et al., 2023).

Herein, we describe several flavonoids including astragalin, kaempferol and its deriatives, quercetin derivatives and some phenolic acids from the water extract of *P. volubilis* leaves and its EA extract (Table 1). In relation to quercetin, its presence has also been observed in an ethanol extract of seed at a concentration of 71.65 ± 0.12 µg/100 g dry seed weight (Kittibunchakul et al., 2023). Apigenin was also identified in the pressed oil either by commercial method or by cold-pressed (Ramos-Escudero et al., 2021). In addition, kaempferol was identified both in the shell and fruit capsule as subproducts of the process to obtain the seed for oil production (Kittibunchakul et al., 2022b; Poomanee et al., 2024) verified that fruit capsule also contains various types of catechins, including (−) and (+) catechin as well as derivatives.

Apigenin and kaempferol with amounts ranging between 2.47–5.82 and 7.62–10.76 µg/100-g dry leaf weight, respectively, were previously identified in *P. volubilis* leaves (Kittibunchakul et al., 2022a). Moreover, it was observed that oven-dried leaves have lower apigenin and kaempferol content when compared to freeze-dried leaves, which could be attributed to degradation of the heat-sensitive flavonoids in the drying process. The presence of kaempferol ranged 0.11 ± 0.0032 g/kg dry leaf weight (Lin et al., 2022a). HPLC analysis also showed that leaves also contain 50.50 ± 4.78 mg/quercetin per Gram dry weight, which may suggest that quercetin is more concentrated in the leaves than the seeds (Wuttisin et al., 2021a; Wuttisin et al., 2021b; Nascimento et al., 2013) also identified the presence of flavonoids derived from kaempferol in leaf extracts.

In relation to antimicrobial activity based on the flavonoids identified in the pressed oil, no significant effect on *Staphylococcus aureus* was observed when compared to the control group using coconut oil. On the other hand, it was observed an effect in preventing the adherence of *S. aureus* cells on keratinocytes (Gonzalez-Aspajo et al., 2015). It was also evaluated the inhibition of bacterial growth using the bacteria: *Escherichia coli*, *Pseudomonas fluorescens*, *Pseudomonas aeruginosa*, *Staphylococcus epidermidis*, *Staphylococcus aureus*, and *Bacillus cereus* and the fungi *Candida albicans*. The *in vitro* results showed no inhibitory activity (Makiej et al., 2024).

In the current study, EA extract from *P. volubilis* leaves water infusion, that was separated using HSCCC, allowed for the isolation and structural characterisation of astragalin. Astragalin has been previously isolated from *Cassia alata, Moringa oleifera, Nelumbo nucifera, Cuscuta* spp., *Radix astragali*, *Morus alba*, and *Eucommia ulmoides*. In relation to its antimicrobial activity, it is effective against skin diseases including eczema, gastric ulcers. Klein-Junior et al. (2013) observed that the treatment with 125 mg/kg of *Polygala cyparissias* St. Hil. and Moquin extracts was efficient to reduce the area of ulcer lesion in mice. These extracts were obtained using acetone or methanol and one of the bioactive molecules identified were astragalin. Although astragalin was not yet tested for *H. pylori*, its aglycone, kaempferol, exhibited an increased growth inhibition halo size compared to the control in concentrations of 0.6 mg/mL and higher. A similar effective growth rate reduction for *H. pylori* grown in culture medium containing kaempferol was also described (Escandón et al., 2016; González et al., 2019; Qing et al., 2023). Kaempferol also produced a protective effect through downregulating secretory genes controlling release of CagA and VacA, which reduced the inflammatory response towards gastric epithelial cells (Yeon et al., 2019). In the current study, we show for the first time that *P. volubilis* tea, it’s EA extract and astragalin exhibit selective bacteriostatic activity against 3 strains of *H. pylori*, including a physiologically relevant clarithromycin-resistant clinical isolate of *H. pylori,* with no inhibitory effect on human AGS gastric epithelial cells. A meta-analysis by Wald-Dickler et al. (2018) of randomised controlled trials has shown no intrinsic superiority of bactericidal compared to bacteriostatic agents in the treatment of clinical bacterial infections. Additional testing using increased numbers of drug sensitive and resistant clinical isolates would further strengthen our data. Future studies should also assess whether the water infusion, EA extract and/or astragalin decrease bacterial load and reduce inflammation in mouse models of *H. pylori* infection to provide a rationale for potential clinical trials.

The molecular docking analysis in this paper sheds light in the potential mechanism of interaction from astragalin ligand to different markers presented at Table 4. To directly assess the role of the target proteins identified by the *in silico* analysis, the extracts should be further evaluated using isogenic mutant strains of *H. pylori* in which the protein of interest has been deleted. Considering, that stomach is a hostile environment to survive due to its pH, however, *H. pylori* have different mechanisms and enzymes involved on its adaptation to survive in this acid pH. For example, the enzymes urease and carbonic anhydrase are important to keep pH neutral in bacteria cytoplasm. The enzyme urease, that is only present in *H. pylori* genome, and this enzyme is formed by two subunits (UreA and UreB) that together with a nickel-responsive regulator (NikR) are important to nickel uptake and important to urease maturation and its function. This function is essential to help *H. pylori* colonization and acclimation in the acid pH. It has been shown that urease and nickel regulator are important targets for drug development. Some drugs used to eradicate this bacterium are based on bismuth, which inhibits the nickel transport and it affect *H. pylori* survival in stomach (Ernst et al., 2005; Rowinska-Zyrek et al., 2010; Salillas and Sancho, 2020).

Moreover, the other marker evaluated by molecular docking was the protein CagA. This protein interacted to other proteins from host cells, and it has been associated to the transition from severe gastritis to the development of carcinoma (Hatakeyama, 2004). Drug development has been searching for different targets that affect the interaction of CagA to different host proteins that has been associated to this transformation (Salillas and Sancho, 2020; Junaid et al., 2019) showed by docking simulation the potential for interaction from CagA (bacteria protein) and ASSP2 protein, and how is important for this protein interaction a peptide mutagenesis to increase the stability of this affinity and consequently reduce the disease severity. The astragalin docking presented here showed a positive potential for affinity, probably reducing the disease severity. However, more studies are important to identify by which mechanism.

The HsrA (homeostatic stress regulator) is an important protein for *H. pylori* viability, hence justifying its choice here as a target for molecular docking with astragalin. This sequence is highly conserved in the Epsilonproteobacteria showing its importance, and its function as transcriptional activator, modulating its own expression, and the expression of other proteins involved in functions as translational, energy metabolism, redox homeostasis. González et al. (2019) search in a library target that can interact to HsrA. They identified 1,120 small molecules that were FDA-approved and off patent. From these molecules, four flavonoids – like apigenin, chrysin, kaempferol and hesperetin – that exhibited antimicrobial activities by binding to HsrA protein and it affected DNA binding from HsrA to other regions important to regulate the expression of crucial genes for energy metabolism, nitrogen metabolism, and redox homeostasis. The molecular docking showed here with astragalin showed interactions with His 66 and Ser 68, a different region as observed by González et al. (2019).

In addition, flavodoxin a small protein is present in some pathogenetic bacterial, but also in human gut microbiota. It is a small electron transfer protein essential to *H. pylori* metabolism. Although, this protein is present in other commensal bacteria, in balance, it has been considered a potential target for drugs development to avoid some side-effects on gut microbiota (Salillas and Sancho, 2020). Here, it was shown that the flavonoid astragalin presented in *P. volubilis* can interact to this protein (Ala 50, Arg 246, Arg 250, Leu 285) probably interfering on its function, consequently being a potential therapy for *H. pylori*.

The results of this paper analysed side by side with the review presented here show the potential of *P. volubilis* for being used as a preventative for *H. pylori*, and astragalin, the flavonoid isolated using HSCCC approach, could be one of the molecules responsible for such activity. This set of interactions underlines the importance of exploring how compounds like astragalin can be used to modulate the pathogenicity of *H. pylori*, offering new perspectives for the development of more effective therapeutic strategies.

## 5 Conclusion

In this study some flavonoids derived from kaempferol were identified in the water infusion of *P. volubilis* leaves using HPLC-ESI-QTOF-MS/MS. Astragalin was further isolated by HSCCC and chemically characterised by ^1^D and ^2^D NMR analysis. In addition, molecular docking for *H. pylori* protein targets and *in vitro* anti-*H. pylori* assays demonstrated that *P. volubilis* water extract, EA extract and astragalin produce an inhibitory effect against clarithromycin-resistant and sensitive strains of *H. pylor*i. Cytotoxicity studies also revealed that the water extract and astragalin did not induce any cytotoxicity against AGS gastric epithelial cells. All this information showed that *P. volubilis* may be a potential plant to be used in the treatment against *H. pylori*. More studies are required to understand the possible mechanisms as well as to isolate and identify other potential pharmacological active flavonoids present in this infusion. In addition, further studies are needed to confirm the safety and efficacy of our *in vitro* results on animals and humans and to evaluate the clinical relevance of these findings in the general population.

## Data Availability

The data is presented in the article and Supplementary Material. Any other question can be made to the corresponding author.
