# Supplementary material for: Plukenetia volubilis leaves as source of anti-Helicobacter pylori agents
Source: Front Pharmacol. 2024 Oct 23;15:1461447. doi: 10.3389/fphar.2024.1461447 (PMC11537943; doi:10.3389/fphar.2024.1461447)
Supplement: Supplementary file 1 [file DataSheet1.docx]

Supplementary material

1.
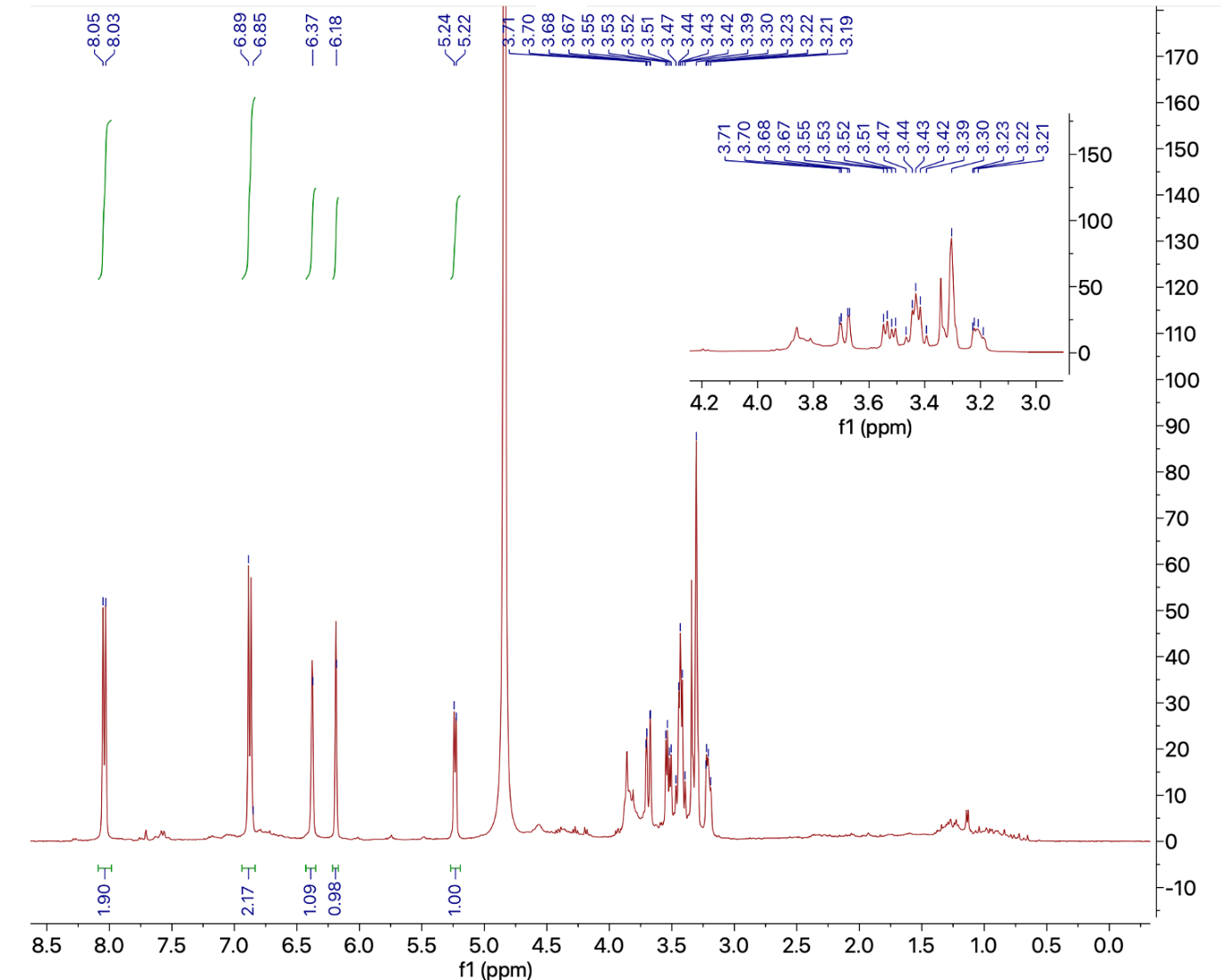

2.
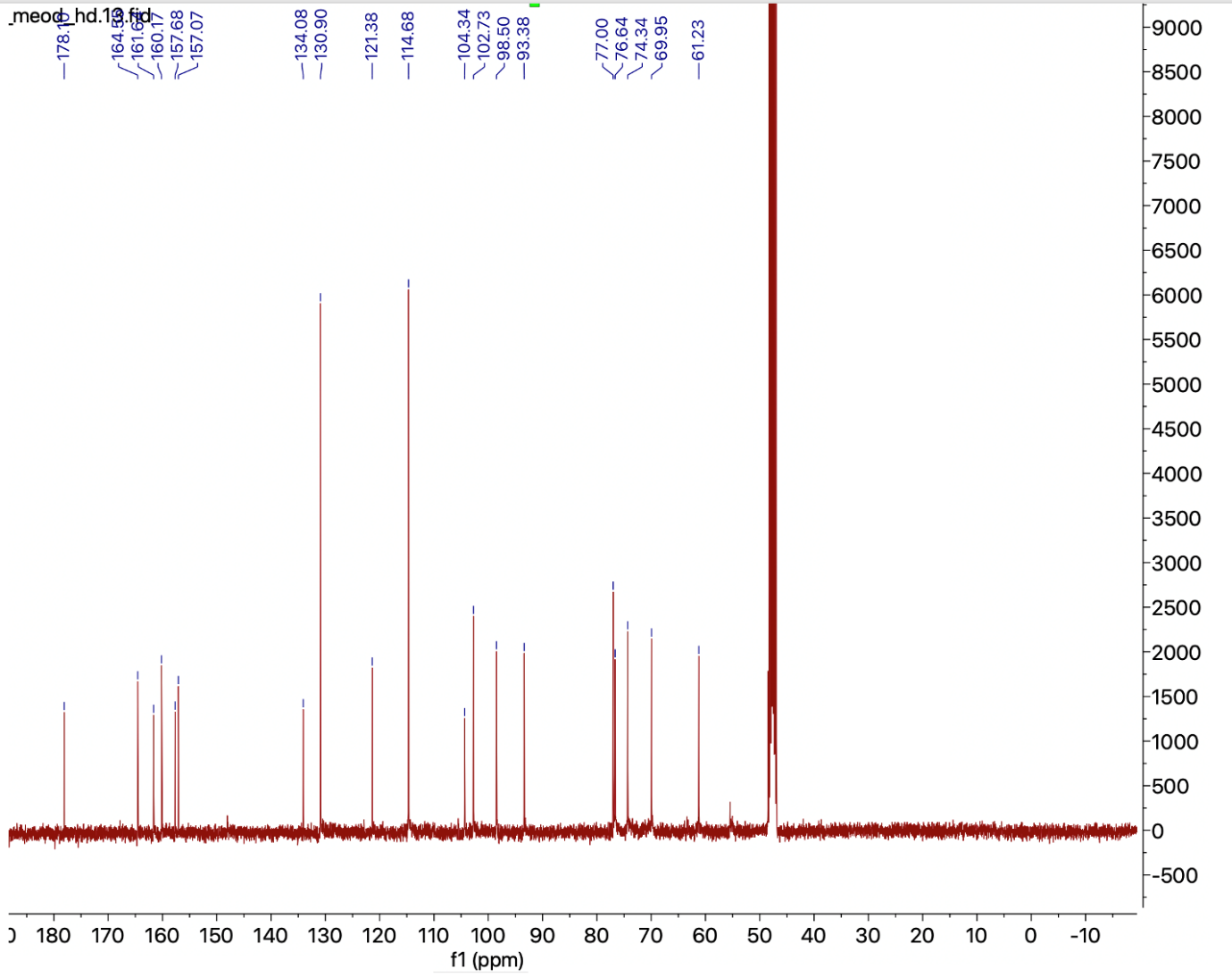

3.
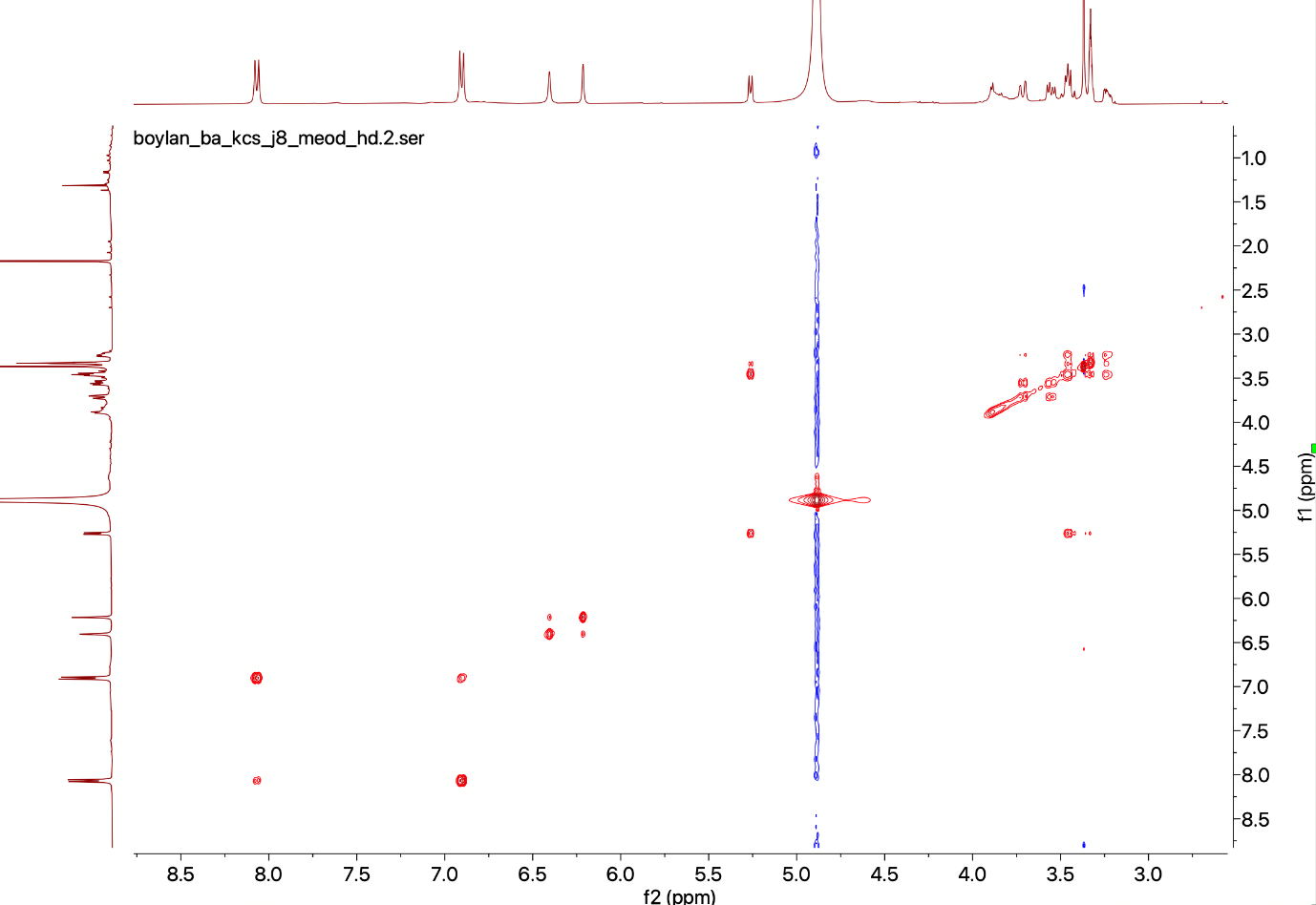

4.
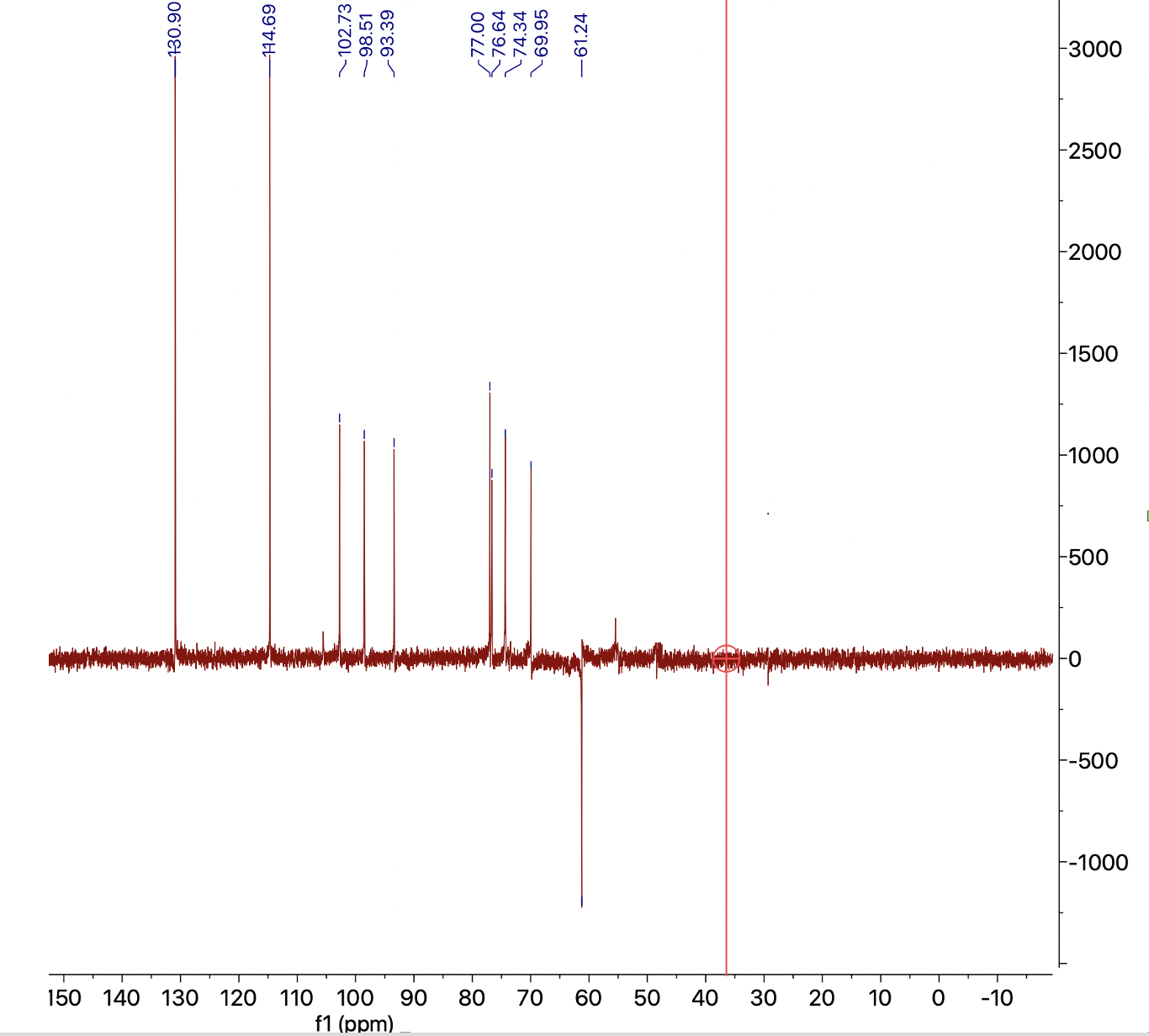

5.
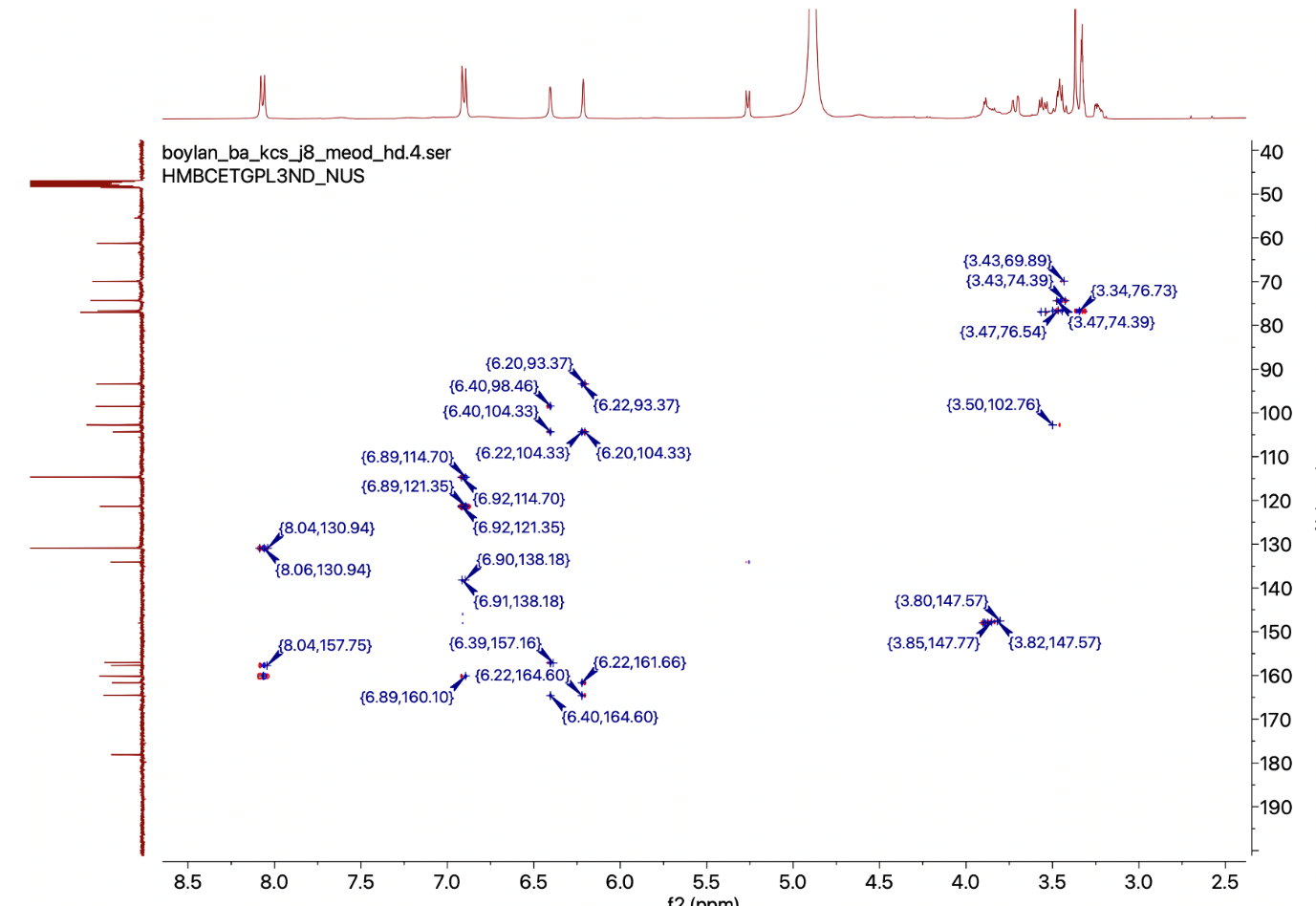

6.
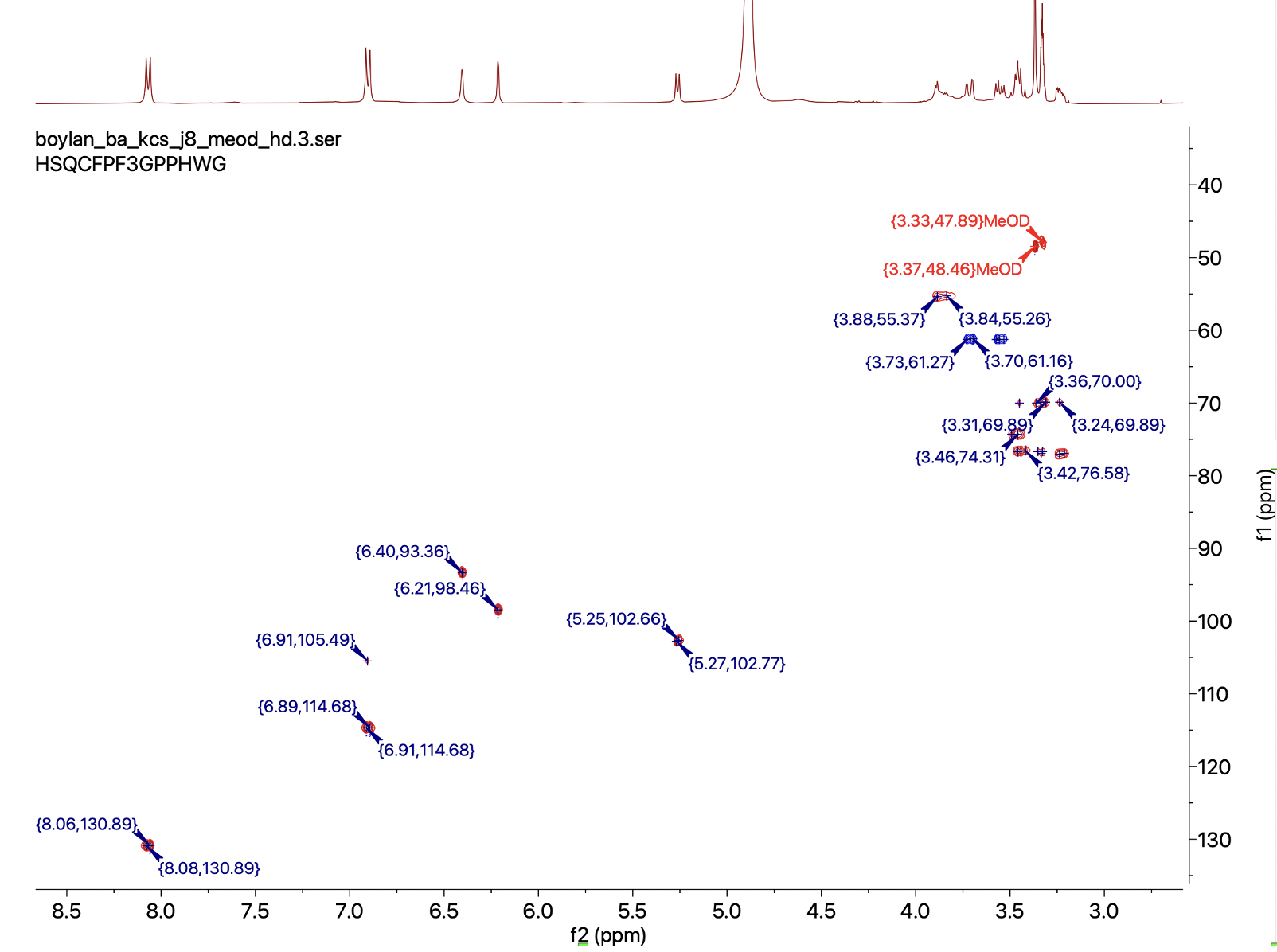


*Supplementary Figure S1 – Astragalin structural analysis* *presented by NMR analysis (a)^1^H-NMR (methanol-d4, 400 MHz), (b) ^13^C-NMR (methanol-d4, 100 MHz), (c) ^1^H-^1^H COSY (d) DEPT135 (e) HMBC (f) HSQC (blue colour shows the ^1^H-^13^C correlation of -CH- and -CH_3_ groups, green colour shows the ^1^H-^13^C correlation of -CH_2_- groups)*

**Supplementary Tables**

Supplementary Table S1 – Sequences used for molecular docking

| Protein | PDB | AlphaFold ID |
| --- | --- | --- |
| NikR | 2WVB | AF-025896-F1 |
| HsrA | — | AF-A0A496FN53-F1 |
| CagA | 4DVY | AF-P55980-F1 |
| BabA | 4ZH0 | AF-Q17SX4-F1 |
| Urease | 1E9Z | AF-P69996-F1 |
| Flavodoxin | — | AF-O25342-F1 |

Supplementary Table S2 – The MS/MS spectra of the assigned compounds from the HPLC-ESI-QTOF-MS/MS analysis

| **No** | **MS/MS spectrum** | **Proposed compound** |
| --- | --- | --- |
| 1 |  | Malic acid |
| 2 |  | Syringic anhydride |
| 3 |  | Citric acid |
| 4 |  | Crotonoside |
| 5 |  | Kaempferol trihexoside |
| 6 |  | Kushenol D |
| 7 |  | Crolaevinoid C |
| 8 |  | Kaempferol trihexoside/kaempferol sophoroside glucoside |
| 9 |  | Kaempferol dihexoside |
| 10 |  | Kaempferol dihexoside |
| 11 |  | Quercetin glucoside, e.g. isoquercitrin/quercetin galactoside |
| 12 |  | Kaempferol-3-O-glucoside (astragalin) |
| 13 |  | Kaempferol glucoside |
| 14 |  | Methyl 3-(3,5-di-tert-butyl-4-hydroxyphenyl)propanoate |
| 15 |  | Crotonpyrone B |
| 16 |  | Crotonolide J |
